# The Combination of a Novel GLUT1 Inhibitor and Cisplatin Synergistically Inhibits Breast Cancer Cell Growth By Enhancing the DNA Damaging Effect and Modulating the Akt/mTOR and MAPK Signaling Pathways

**DOI:** 10.3389/fphar.2022.879748

**Published:** 2022-05-19

**Authors:** Hao-Cheng Weng, Chieh-Ju Sung, Jui-Ling Hsu, Wohn-Jenn Leu, Jih-Hwa Guh, Fan-Lu Kung, Lih-Ching Hsu

**Affiliations:** ^1^ School of Pharmacy, National Taiwan University, Taipei, Taiwan; ^2^ Department of Pharmacy, New Taipei Municipal TuCheng Hospital, Chang Gung Memorial Hospital, New Taipei City, Taiwan

**Keywords:** breast cancer, cisplatin, GLUT1 inhibitor, oxidative stress, DNA damage, Akt/mTOR and MAPK signaling pathways

## Abstract

Breast cancer is the most prevalent cancer and the second leading cause of cancer death in women. Cisplatin is a commonly used chemotherapeutic drug for breast cancer treatment. Owing to serious side effects, the combination of cisplatin with other drugs is an effective strategy to simultaneously reduce side effects and increase the anticancer efficacy. GLUT1 is an emerging target for cancer treatment since cancer cells usually consume more glucose, a phenomenon called the Warburg effect. In this study, we found that the combination of cisplatin and a novel GLUT1 inhibitor #43 identified from our previous high-throughput screening exerted a synergistic anticancer effect in MCF-7 and MDA-MB-231 breast cancer cells. Mechanism studies in MCF-7 cells revealed that combination of cisplatin and #43 significantly induced apoptosis, intracellular reactive oxygen species, and loss of mitochondrial membrane potential. Furthermore, #43 enhanced the DNA damaging effect of cisplatin. Akt/mTOR downstream signaling and the ERK signaling pathway usually involved in cell growth and survival were inhibited by the combination treatment. On the other hand, phosphorylation of p38 and JNK, which may be associated with apoptosis, was induced by the combination treatment. Altogether, our data indicate that oxidative stress, DNA damage, the Akt/mTOR and MAPK signaling pathways, and apoptosis may be involved in the synergism of cisplatin and #43 in breast cancer cells.

## Introduction

Breast cancer is a very common malignancy. According to the survey of the American Cancer Society, breast cancer is the most frequently diagnosed cancer and the second leading cause of cancer death in women (Siegel et al., 2022). Hormone therapy is usually used for estrogen receptor-positive and progesterone receptor-positive breast cancer, targeted therapy can be used for HER2-positive breast cancer, while chemotherapeutic drugs, such as anthracyclines, taxanes, platinum agents and vinca alkaloids, are commonly used for the treatment of triple-negative breast cancer and advanced breast cancer. The combination of two or more drugs is more effective than single drug treatment.

Cancer cells preferentially use glycolysis for energy production and require more glucose, a phenomenon called the Warburg effect (Vander Heiden et al., 2009). Thus, targeting the glycolytic pathway to interfere with both the energy production and anabolic reactions is an attractive strategy to fight cancer. Enzymes participating in glycolysis, such as hexokinase, phosphofructokinase, pyruvate dehydrogenase kinase, lactate dehydrogenase, and pyruvate kinase, have become potential targets for the development of anticancer drugs (Gill et al., 2016). In addition, glucose transporter GLUT1 is overexpressed in various malignant tumors and considered a potential target for cancer therapy. Several GLUT1 inhibitors have been identified and may have applications in cancer treatment (Granchi et al., 2016; Reckzeh and Waldmann, 2020). For example, WZB117 was identified as a potent synthetic GLUT1 inhibitor with good activity against non-small cell lung cancer (NSCLC) cells both *in vitro and in vivo* (Liu et al., 2012). However, like drugs targeting glycolysis (Pelicano et al., 2006), the anticancer efficacy of GLUT1 inhibitors may not be sufficient as single agents, and proper combination with other anticancer drugs may be required to effectively kill cancer cells.

Cisplatin, the first platinum-based anticancer drug approved by the FDA, is a frequently used chemotherapeutic drug (Kelland, 2007). It has been reported that cisplatin formed intrastrand or interstrand crosslinks with DNA, causing DNA replication errors and DNA damage, ultimately leading to apoptosis in cancer cells. Furthermore, reactive oxygen species (ROS), the calcium signaling, mitogen-activated protein kinase (MAPK) and PI3K/Akt pathways may also be involved in the cytotoxicity of cisplatin. In spite of the strong antineoplastic effects, cisplatin may cause serious side effects, such as nephrotoxicity, neurotoxicity, ototoxicity, hepatotoxicity, and cardiotoxicity that may restrict its usage (Florea and Büsselberg, 2011; Dasari and Tchounwou, 2014). Therefore, combination treatment is a well-accepted strategy to enhance the anticancer activities and reduce the undesirable side effects by lowering the dosage. For instance, carboplatin, a cisplatin analog, combined with paclitaxel is commonly used for treatment of advanced breast cancer.

The DNA damage response (DDR) is essential for maintaining genome integrity. DNA damage can emerge from exogenous stresses caused by ionizing radiation and chemotherapeutic drugs or from endogenous stresses, for example, ROS and DNA replication errors. DNA double-strand breaks (DSBs) are the most detrimental DNA damage that may cause cell death if DNA repair fails. Error-free homologous recombination (HR) and error-prone non-homologous end joining (NHEJ) are the two major mechanisms for DSB repair. During S/G2 phases of the cell cycle, DSBs can be recognized by the MRE11/RAD50/NBS1 (MRN) complex and subsequently ATM is activated and Rad51 recombinase is recruited to repair DSBs via HR. NHEJ functioning throughout the interphase is initiated by binding of the Ku70/Ku80 complex to DSBs, leading to the recruitment and activation of DNA-dependent protein kinase catalytic subunit (DNA-PKcs). A histone H2A variant (H2AX) is rapidly phosphorylated by activated ATM or DNA-PKcs, and the phosphorylated H2AX (γ-H2AX) is a marker for DNA damage. Activation of checkpoint kinases Chk1 and Chk2, along with the downstream signaling through p53 and p21 causes cell cycle arrest for DNA repair, or results in apoptosis if DNA damage is beyond repair (Bohgaki et al., 2010).

Oxidative stress arises as the homeostasis between intrinsic anti-oxidants and ROS is interrupted and excess intracellular ROS may cause damage to DNA, lipid, and protein, leading to loss of physiological functions, and ultimately cell death. ROS comprise superoxide anion, hydrogen peroxide, and hydroxyl ion, and can be generated under a lot of physiological conditions, such as the electron transfer chain of oxidative phosphorylation, and reactions catalyzed by NADPH oxidases and cytochrome P450, or by some anticancer agents, such as cisplatin (Burton and Jauniaux, 2011; Florea and Büsselberg, 2011).

Programmed cell death is a phenomenon of cells committing suicide mediated by intracellular signaling and plays a crucial role in developmental processes, physiological and pathological conditions. Apoptosis is the major type of programmed cell death which may occur in response to stresses, such as severe DNA damage. Apoptosis can be classified into two main pathways: the extrinsic pathway and the intrinsic pathway which are mediated by activation of caspase-8 and caspase-9, respectively, followed by activation of a series of downstream caspases, ultimately leading to apoptosis. PARP is cleaved by caspases and commonly used as a marker for apoptosis. The intrinsic pathway is regulated by the Bcl-2 family proteins, including anti-apoptotic Bcl-2, Bcl-x, and Bcl-xL, and pro-apoptotic Bax, Bid, and Bak (Elmore, 2007). Moreover, it has been demonstrated that p53, a tumor suppressor protein, not only increases the levels of pro-apoptotic proteins, but also antagonizes anti-apoptotic protein functions to regulate apoptosis (Hemann and Lowe, 2006).

We reported previously that WZB117 in combination with an allosteric Akt inhibitor MK-2206 synergistically inhibited the growth of breast cancer cells by downregulating Akt signaling and inducing DNA damage (Li et al., 2019). To search for novel inhibitors of glucose transporters as potential anticancer agents, we conducted high-throughput virtual screening using the National Cancer Institute (NCI) chemical library containing more than 140,000 compounds based on the crystal structure of hGLUT1, which is usually overexpressed in tumor cells. The top 75 hits from virtual screening were then evaluated by cell-based glucose uptake assays including 2-NBDG uptake assay and Glucose Uptake-Glo™ assay, and four novel GLUT1 inhibitors were identified. Among them, #43 showed the best GLUT1 inhibitory effect in MCF-7 breast cancer cells [Hung et al., manuscript in preparation]. Here we report that #43 enhances the cytotoxic effect of cisplatin against breast cancer cells by augmenting its DNA damaging effect. The combination of #43 and cisplatin also compromises the Akt/mTOR and mitogen-activated protein kinase (MAPK) signaling pathways.

## Materials and Methods

### Chemicals

Cisplatin, 2′,7′-dichlorodihydrofluorescein diacetate (DCFH-DA), Sulforhodamine B (SRB) and crystal violet were purchased from Sigma Aldrich (St. Louis, MO) and #43 was obtained from the NCI Developmental Therapeutics Program. MK-2206 was purchased from BioVision (Mountain View, CA). 3-(4,5-Dimethylthiazol-2-yl)-2,5-diphenyltetrazolium bromide (MTT), JC-1 dye, 2-NBDG and propidium iodide (PI) were obtained from Invitrogen Life Technologies (Carlsbad, CA). U0126 was purchased from Cell Signaling Technologies (Boston, MA). N-acetylcysteine (NAC) was purchased from MedChemExpress (Monmouth Junction, NJ). Stock solutions of #43, DCFH-DA, MK-2206 and U0126 were prepared in DMSO, cisplatin and MTT were dissolved in phosphate-buffered saline (PBS), SRB solution was prepared in 1% acetic acid, 2-NBDG, PI and NAC were dissolved in water, and crystal violet was dissolved in 20% methanol.

### Cell Culture, Cell Viability Assay and Combination Index Analysis

Human breast cancer cells MCF-7 (originally obtained from Michigan Cancer Foundation) and MDA-MB-231 (purchased from ATCC) were cultured in high-glucose Dulbecco’s Modified Eagle’s Medium supplemented with 10% FBS (v/v), 2 mM L-glutamine, and antibiotics (100 units/ml penicillin, 100 μg/ml streptomycin and 0.25 μg/ml amphotericin B) at 37°C in a humidified 5% CO_2_ atmosphere, and subcultured when reaching 80–90% confluence. For cell viability assay, cells were seeded into 96-well plates (4,000–5,000 cells/well) and treated with indicated concentrations of cisplatin and/or #43 for 72 h, followed by the MTT or SRB assay as previously described (Huang et al., 2020). The combination index (CI) was calculated by Compusyn software (Chou and Talalay, 1984; Chou, 2006). CI values <1, = 1, and >1 represent synergistic, additive, and antagonistic effect, respectively.

### Colony Formation Assay

Cells were seeded into 6-well plates (1,000 cells/well) overnight and then treated with 10 μM cisplatin for 1 h and then drug-free medium for 10–12 days (CDDP), 10 or 20 μM #43 for 10–12 days (#43), or 10 μM cisplatin combined with 10 or 20 μM #43 for 1 h and then 10 or 20 μM #43 for 10–12 days (CDDP+#43). Colonies were rinsed with PBS, stained with 0.4% crystal violet in 20% methanol for 30 min, rinsed with tap water, and then air-dried. Colonies with at least 50 cells were counted and colony formation was calculated as the percentage relative to the vehicle control.

### 2-NBDG Uptake Assay

Cells were seeded into 12-well plates (2.5 × 10^5^ cells/well) and cultured overnight. Cells were then rinsed with PBS and incubated with indicated drugs in the presence of 200 μM 2-NBDG in PBS for 1.5 h. After harvested by trypsinization, cells were resuspended in PBS and subjected to flow cytometric analysis using FACSCalibur (BD Biosciences, San Jose, CA) and the results were analyzed by FlowJo software (Tree Star Inc., Ashland, OR). The geomean of cell background fluorescence (without 2-NBDG) was subtracted from the geomean of fluorescence in the presence of 2-NBDG, and the relative 2-NBDG uptake was calculated using the vehicle control group as 100%.

### Small Interfering RNA Transfection and Cell Viability Assay

Cells were seeded into 96-well plates (5,000 cells/well) overnight and transfected with siRNAs using the Lipofectamine 2000 transfection reagent. *GLUT1* siRNA (SMARTpool) was obtained from Dharmacon, and negative control siRNA (sc-37007) was obtained from Santa Cruz Biotechnology (Santa Cruz, CA). Two pmoles of siRNA in 100 μl of antibiotic-free culture medium were used for each transfection in a 96-well. After transfection, cells were treated with indicated drugs for 72 h and cell viability was measured by the MTT assay. The efficiency of *GLUT1* knockdown by siRNA was more than 50% determined by Western blot analysis as previously reported (Li et al., 2019).

### Cell Cycle Analysis

Cells were seeded into 6-well plates (3 × 10^5^ cells/well) and treated with indicated drugs for 24 or 48 h. Subsequently, cells were harvested by trypsinization and fixed overnight with 70% (v/v) ethanol at −20°C. After centrifugation at 500 × *g* for 5 min at 4°C, cells were stained with PI solution for 30 min in the dark and subjected to flow cytometric analysis using FACSCalibur and the results were analyzed by FlowJo software.

### Annexin V-FITC/PI Double Staining

Annexin V-FITC/PI double staining was utilized to detect apoptotic cells. Briefly, cells were seeded into 6-well plates (2.5 × 10^5^ cells/well), harvested by trypsinization after drug treatment, washed with cold PBS and resuspended in 1 × Annexin V-FITC binding buffer (10 mM HEPES pH 7.4, 140 mM NaCl, and 2.5 mM CaCl_2_). Annexin V Apoptosis Detection kit (sc-4252 AK, Santa Cruz Biotechnology) was used to stain the cells, which were then analyzed immediately by flow cytometry using FACSCalibur and results were analyzed by FlowJo software.

### Measurement of ROS

Cells were seeded into a 12-well plate (1.5 × 10^5^ cells/well). Cells pretreated with or without 2 mM antioxidant NAC for 1 h were then incubated with indicated drugs for 30 min or 24 h. DCFH-DA (10 μM) was added to the cells 30 min before termination of the incubation, and cells were harvested by trypsinization, resuspended in cold PBS and then subjected to flow cytometric analysis using FACSCalibur and the results were analyzed by FlowJo software.

### Measurement of Mitochondrial Membrane Potential

Cells were seeded into 6-well plates (2.5 × 10^5^ cells/well) and treated with indicated drugs for 48 h. Before the treatment termination, cells were stained with JC-1 dye (5 μg/ml) for 30 min before the termination of drug treatment and harvested by trypsinization, resuspended in PBS and subjected to flow cytometry using FACSCalibur and the results were analyzed by FlowJo software.

### Western Blot Analysis

Cells were harvested by trypsinization after the indicated treatment, washed with cold PBS and lysed with lysis buffer containing 10 mM Tris-HCl pH 7.5, 150 mM NaCl, 1% Triton X-100, 1 mM EDTA, 1 mM EGTA, 50 mM NaF, 1 mM Na_3_VO_4_ and protease inhibitor cocktail (Roche Diagnostics, Indianapolis, IN) for 30 min on ice and centrifuged at 17,000 × *g* for 15 min at 4°C. After centrifugation, supernatants were collected, the protein concentration was determined using the Bio-Rad protein assay kit (Bio-Rad Laboratories, Hercules, CA), and lysates were denatured in sample buffer (62.5 mM Tris-HCl pH 6.8, 10% glycerol, 2% SDS, 0.005% bromophenol blue, 1.4% β-mercaptoethanol) for 10 min at 95°C. Lysates containing 10 μg proteins were subjected to 10% SDS-PAGE, transferred to PVDF membrane, followed by Western blot analysis. Image detection and quantification were performed using the ChemiDoc XRS system and Image Lab software (Bio-Rad Laboratories, Hercules, CA). Primary antibodies used were Bax, Bid, p-Chk1 (S345), p-Chk2 (T68), Ku80, phospho-Akt (p-Akt, S473), Akt, p-mTOR (S2448), mTOR, p-p70S6K (T389), p70S6K, p-4EBP1 (T37/46), 4EBP1, p-MEK1/2 (S217/221), p-ERK1/2 (T202/Y204), ERK1/2, p-p38 (T180/Y182), p38, p-c-Jun (Cell Signaling Technology, Boston, MA), p-JNK (Y185/223), JNK (Abcam PLC, Inc., Cambridge, MA), PARP, Bcl-2, Chk1, Chk2, Rad51, c-myc (Santa Cruz Biotechnology), p-p53, p53, p21, and γ-tubulin (Sigma-Aldrich, St. Louis, MO). Secondary antibodies used were HRP-conjugated goat anti-mouse and anti-rabbit IgGs (Cell Signaling Technology). γ-tubulin was used as a loading control.

### Immunofluorescence Staining

Cells were seeded into 8-well chamber slides (2.5 × 10^4^ cells/well), and treated with indicated drugs for 12 h. Cells were then fixed on ice for 30 min in PBS-buffered 3% paraformaldehyde/2% sucrose solution, and permeabilized in 0.5% Triton X-100, 20 mM HEPES, pH 7.4, 50 mM NaCl, 3 mM MgCl_2_, and 300 mM sucrose at room temperature for 5 min, and then subjected to immunofluorescence staining (Li et al., 2019). Primary antibody used was γ-H2AX (1:1,000 dilution, Millipore, Billerica, MA) with Texas Red-conjugated goat anti-mouse IgG (1:200 dilution) as the secondary antibody. DAPI was used for nuclear counter staining, and slides were mounted with antifade (Invitrogen, Carlsbad, CA). Images were acquired on a fluorescence microscope (Carl Zeiss GmbH, Jena, Germany) with a 40× objective. At least 100 cells were counted for each sample, and the percentage of γ-H2AX positive cells was calculated.

### HR Assay

Cells were seeded in 6-well plates (2-4 × 10^5^ cells/well) and transfected with pDR-GFP and pCMV-I-*Sce*I. One day after transfection, cells were treated with DMSO, 20 μM cisplatin, 20 μM #43, or the combination of cisplatin and #43 for 24 h and harvested for flow cytometric analysis using FACSCalibur on a two-dimensional dot plot of the GFP fluorescence (FL1) and cell autofluorescence (FL2) as described (Li et al., 2019).

### NHEJ Assay

Cells were seeded in 6-well plates (3 × 10^5^ cells/well) and transfected with the pGL3-Control plasmid linearized by *Hin*dIII digestion. One day after transfection, cells were treated with DMSO, 20 μM cisplatin, 20 μM #43, or the combination of cisplatin and #43 for 24 h, and then subjected to the luciferase assay using a Luciferase Assay System (Promega, Madison, WI) as previously described (Li et al., 2019). The *Hin*dIII restriction enzyme cuts the pGL3-Control plasmid between the SV40 promoter and the *luc*+ coding sequence. Luminescence can only be detected when the linearized plasmid is re-ligated by NHEJ and luciferase is expressed inside the cell. Luminescence in lysate of pGL3-Basic (lacking an upstream promoter to drive luciferase expression) transfected cells was measured and served as the background signal.

### Data Analysis

Data are presented as mean ± SEM of at least three independent experiments. Statistical analysis was performed by one-way analysis of variance (ANOVA) followed by Bonferroni *t*-test for multiple groups or two-tailed Student’s *t*-test for comparison of two groups. *P*-values less than 0.05 were considered statistically significant. All data analyses were performed with GraphPad Prism 6 (GraphPad Software Inc., La Jolla, CA).

## Results

### Cisplatin and #43 Synergistically Inhibit the Growth of MCF-7 and MDA-MB-231 Cells

**FIGURE 1:**
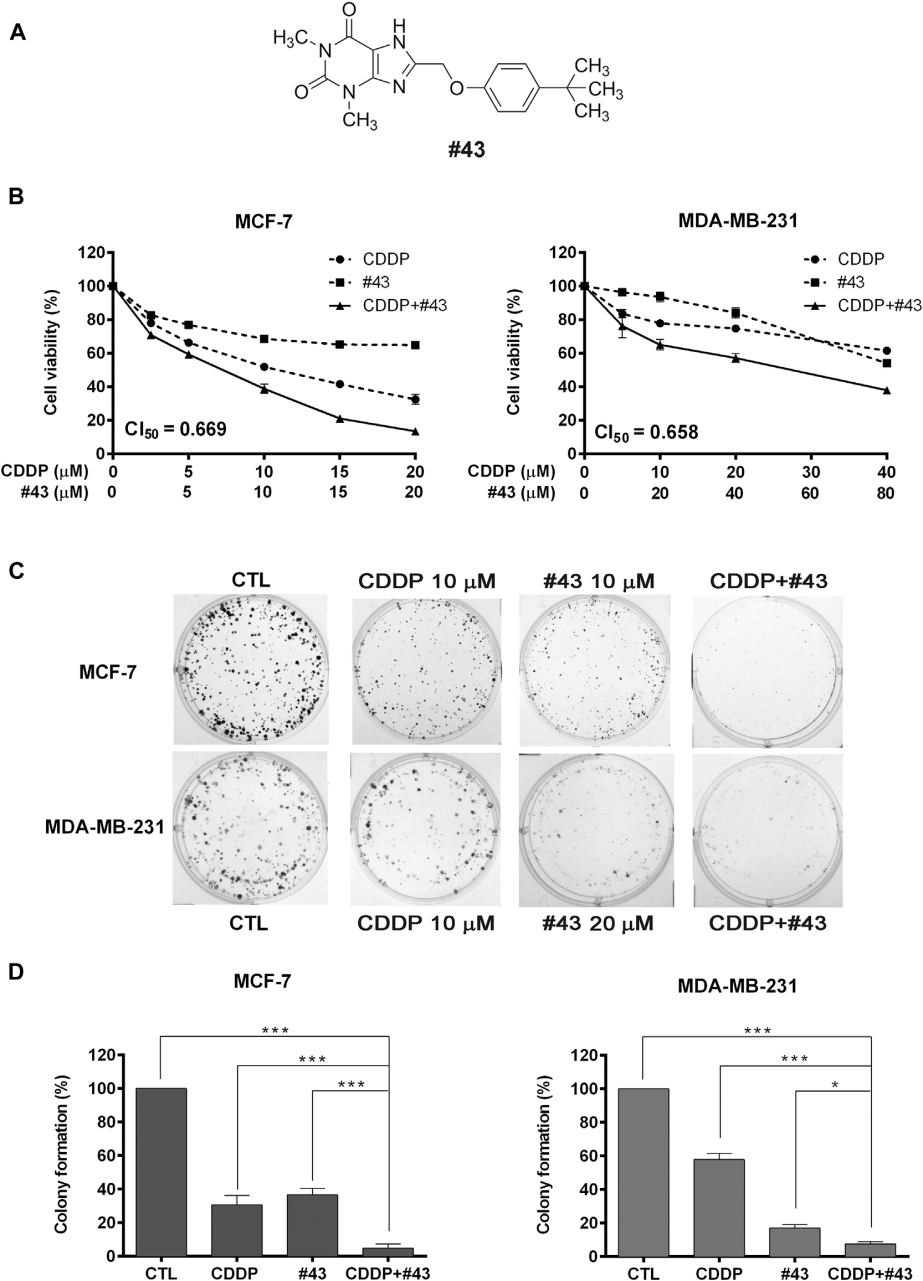
Effects of cisplatin and #43 on growth inhibition and clonogenicity in MCF-7 and MDA-MB-231 cells. **(A)** Structure of #43. **(B)** Dose-response curves and CI_50_ values of MCF-7 (left panel) and MDA-MB-231 cells (right panel) treated with cisplatin (CDDP) and #43 either alone or in combination for 72 h. The MTT assay was used to measure the cell viability. **(C)** Representative images of colony formation assay. MCF-7 and MDA-MB-231 cells were treated with indicated concentrations of cisplatin for 1 h and/or #43 for 10–12 days. **(D)** Quantitative results of colony formation assay. Data are presented as mean ± SEM of at least three independent experiments. *, *p* < 0.05; ***, *p* < 0.001 by one-way ANOVA followed by Bonferroni *t*-test.

We reported previously that a GLUT1 inhibitor WZB117 displayed a synergistic anticancer effect against MCF-7 and MDA-MB-231 breast cancer cells when combined with a potent Akt inhibitor MK-2206 (Li et al., 2019). Through virtual screening and then cell-based assays, we have identified a novel GLUT1 inhibitor #43 [NSC36525; IUPAC name: 8-[(4-tert-butylphenoxy)methyl]-1,3-dimethyl-7H-purine-2,6-dione, structure is shown in Figure 1A] from the NCI chemical library with potency comparable to phloretin and WZB117 (Hung et al., manuscript in preparation), and sought to investigate its potential application in combination with other anticancer agents. Among several compounds tested in a preliminary study, including MK-2206, BEZ-235, cisplatin, and doxorubicin in several cancer cell lines including MCF-7 breast cancer cells, cisplatin consistently showed a better combinatorial effect with #43 (Supplementary Figure S1). Therefore, the combination of cisplatin and #43 was further investigated in breast cancer cells.

In the preliminary study, the combination of 10 μM #43 and 10 μM cisplatin showed a good combinatorial effect in MCF-7 cells. Thus, to determine the synergism, MCF-7 cells were treated with 0–20 μM of cisplatin and #43 either alone or in combination at a 1:1 molar ratio for 72 h, and the MTT assay was subsequently used to evaluate the cell viability. The dose-response curves are shown in Figure 1B, left panel. Combination index (CI), an indicator of drug interactions, was then calculated from the cell viability data using CompuSyn software. The CI value at 50% growth inhibition (CI_50_) of the combination of cisplatin and #43 in MCF-7 cells was 0.669, indicating a synergistic effect.

MDA-MB-231 triple-negative breast cancer cells were treated with 5 or 10 μM of cisplatin in combination with 10 μM of #43 for 72 h in a preliminary study, and the MTT assay revealed that 10 μM of #43 enhanced the growth inhibitory effect of both 5 and 10 μM cisplatin; however, the combinatorial effect of 5 μM cisplatin and 10 μM #43 was slightly better (Supplementary Figure S2). Therefore, a 1:2 molar ratio was chosen, and MDA-MB-231 cells were treated with 0–40 μM of cisplatin and 0–80 μM #43 either alone or in combination for 72 h. As illustrated in Figure 1B, right panel, this combination was also synergistic with a CI_50_ value of 0.658. Similar synergistic effect was observed when the SRB assay was used to measure cell viability (Supplementary Figure S3).

The colony formation assay was conducted to evaluate the long-term growth inhibitory effect. Cells were treated with 10 μM of cisplatin or 10 μM (MCF-7) or 20 μM (MDA-MB-231) of #43 either alone or in combination for 1 h, and then cisplatin was washed off while #43 was included for continuing culture for 10–12 days. Representative colony formation images are shown in Figure 1C and quantitative data are shown in Figure 1D. Cisplatin (27.30 ± 5.00% colony formation in MCF-7 cells, 57.9 ± 3.51% in MDA-MB-231 cells) or #43 (32.33 ± 1.43% in MCF-7 cells, 17.1 ± 2.06% in MDA-MB-231 cells) alone inhibited clonogenic growth compared to the untreated control (100%), and the combination (2.35 ± 0.63% in MCF-7 cells, 7.55 ± 1.29% in MDA-MB-231 cells) significantly improved the clonogenic inhibitory effect of either drug alone.

Taken together, these results indicated that the combination of cisplatin and #43 synergistically inhibited the growth of MCF-7 and MDA-MB-231 cells.

### #43 Significantly Inhibits Glucose Uptake

**FIGURE 2:**
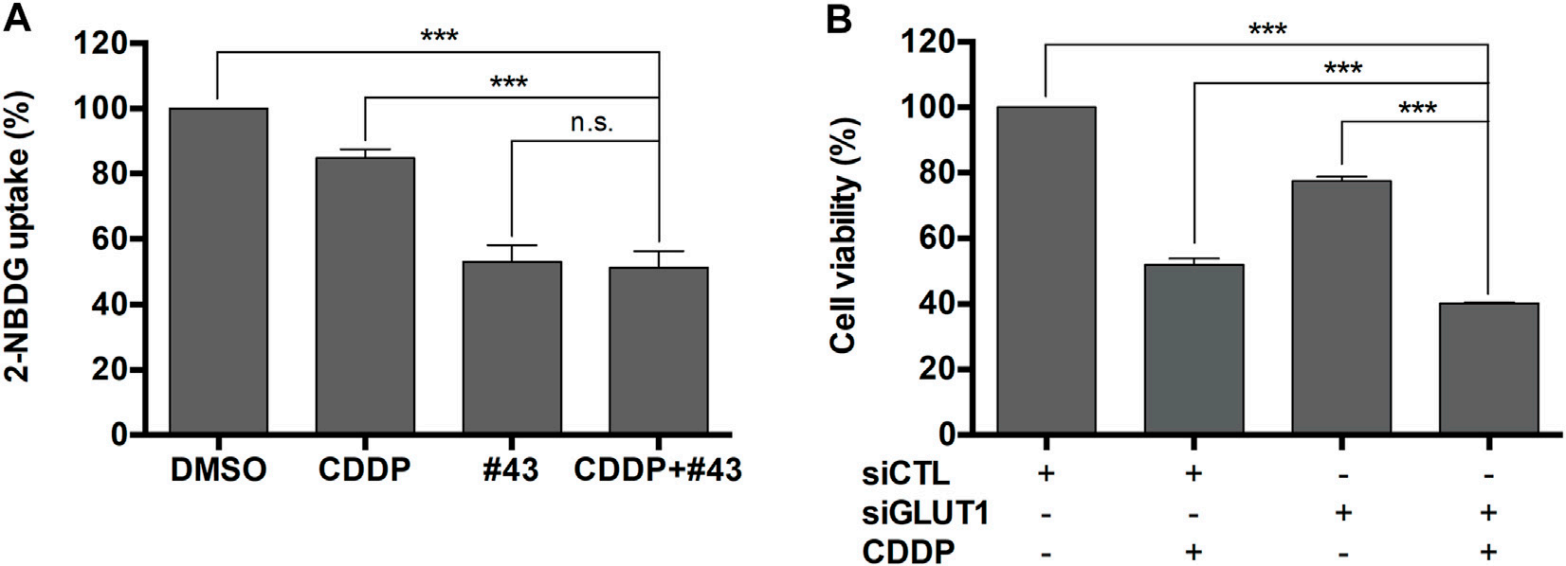
Inhibition of glucose uptake by #43 in MCF-7 cells. **(A)** Quantitative results of 2-NBDG uptake assay. Cells were treated with 20 μM cisplatin and/or 20 μM #43 in PBS along with 200 μM 2-NBDG for 1.5 h, and subjected to flow cytometric analysis. 2-NBDG uptake was calculated from the geomean of 2-NBDG fluorescence in the treated group relative to the DMSO vehicle control. **(B)** Knockdown of *GLUT1* enhanced the growth inhibitory effect of cisplatin. MCF-7 cells were transfected with scrambled siRNA (siCTL) or *GLUT1* siRNA (siGLUT1), and then treated with 10 μM cisplatin for 72 h. Cell viability was measured by the MTT assay. Data are presented as mean ± SEM of at least three independent experiments. ***, *p* < 0.001; n. s., not significant (*p* > 0.05) by one-way ANOVA followed by Bonferroni *t*-test.

MCF-7 cells were then used to investigate the underlying mechanisms of the synergism. #43 was one of the most potent GLUT1 inhibitors identified from our previous screening of compounds in the NCI chemical library. Therefore, 2-NBDG uptake assay followed by flow cytometric analysis was conducted to determine the effect of cisplatin and #43 on glucose uptake. The results showed that both 20 μM #43 (53.04 ± 5.06%) and 20 μM #43 combined with 20 μM cisplatin (51.21 ± 5.10%) significantly inhibited glucose uptake. Interestingly, 20 μM cisplatin alone also slightly suppressed glucose uptake (84.93 ± 2.58%), but there was no statistical significance compared to the DMSO control (100%) (Figure 2A).

To verify whether GLUT1 inhibition was involved in the growth inhibitory effect of the combination of cisplatin and #43, MCF-7 cells were transfected with scrambled control siRNA or *GLUT1* siRNA to knock down *GLUT1* expression as previously reported (Li et al., 2019) and then treated with 10 μM cisplatin for 72 h followed by the MTT assay. As illustrated in Figure 2B, the viability of MCF-7 cells transfected with scrambled control siRNA and treated with cisplatin was reduced to 51.87 ± 1.98% relative to the untreated control siRNA transfected cells, while cells transfected with GLUT1 siRNA showed 77.45 ± 1.44% viability which was further reduced to 40.17 ± 0.24% in the presence of cisplatin. The dose-effect curve and normalized isobologram derived from the data set are illustrated in Supplementary Figure S4. Thus, cisplatin combined with *GLUT1* knockdown further enhanced the growth inhibitory effect of each other, suggesting that GLUT1 inhibition may at least in part contribute to the synergism between cisplatin and #43.

### Effects of Cisplatin and #43 on Cell Cycle Progression

**FIGURE 3:**
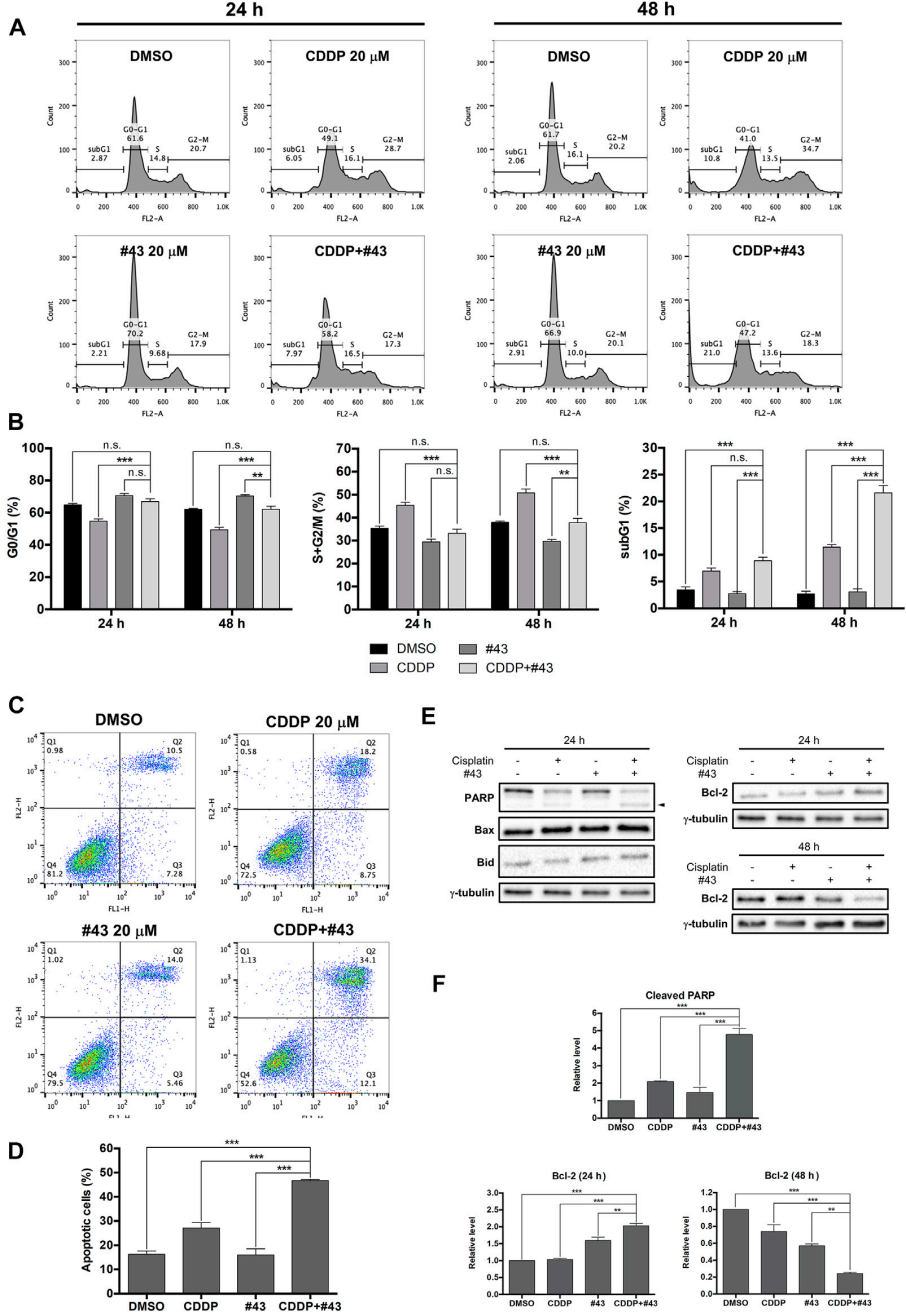
Effects of cisplatin and #43 on cell cycle progression and apoptosis in MCF-7 cells. **(A)** Representative histograms of cell cycle analysis. Cells were treated with 20 μM cisplatin and/or 20 μM #43 for 24 and 48 h, followed by PI staining and flow cytometric analysis. **(B)** Quantitative results of G0/G1, S + G2/M, and subG1 populations. **(C)** Dot plots of Annexin V-FITC/PI double staining after 48 h of 20 μM cisplatin and/or 20 μM #43 treatment. **(D)** Quantitative results of Annexin V-FITC/PI flow cytometric analysis. Cells in Q2 and Q3 were calculated as apoptotic cells. **(E)** Western blot analysis of apoptosis-related proteins. Cleaved PARP is marked with an arrowhead. **(F)** Quantitative results of Western blot analysis. Cells were treated with 20 μM cisplatin and/or 20 μM #43 for 24 or 48 h and harvested for Western blot analysis. The relative protein levels were quantified by Image Lab software and γ-tubulin was used as the loading control. Data are presented as mean ± SEM of at least three independent experiments. *, *p* < 0.05; **, *p* < 0.01; ***, *p* < 0.001; n. s., not significant (*p* > 0.05) by one-way ANOVA followed by Bonferroni *t*-test.

MCF-7 cells were treated with 20 μM cisplatin and 20 μM #43 either alone or in combination for 24 or 48 h followed by PI staining and flow cytometric analysis. Representative histograms are illustrated in Figure 3A and quantitative results are shown in Figure 3B. Cell cycle distribution in G0/G1, S and G2/M was calculated by excluding the subG1 population. #43 significantly increased (70.62 ± 1.22% at 24 h; 70.32 ± 0.18% at 48 h), while cisplatin decreased (54.65 ± 1.37% at 24 h; 49.20 ± 1.62% at 48 h) G0/G1 cells compared to the DMSO control (64.69 ± 0.98% at 24 h; 62.03 ± 0.58% at 48 h). In contrast, cisplatin significantly increased (45.35 ± 1.37% at 24 h; 50.80 ± 1.62% at 48 h) while #43 decreased (29.38 ± 1.22% at 24 h; 29.68 ± 0.81% at 48 h) S and G2/M cells compared to the DMSO control (35.31 ± 0.98% at 24 h; 37.97 ± 0.58% at 48 h). Interestingly, these effects were somewhat attenuated in the combination treatment. Cisplatin increased the subG1 population, and the combination with #43 further enhanced this effect, suggesting an induction of apoptosis. After 48 h of combination treatment, the subG1 population (21.60 ± 1.36%) was markedly increased compared to either cisplatin (11.43 ± 0.49%) or #43 (3.09 ± 0.55%) alone (*p* < 0.001), supporting a synergistic cytotoxic effect.

### #43 Enhances the Apoptotic Effect of Cisplatin

To further confirmed that the combination of cisplatin and #43 synergistically induced apoptosis, MCF-7 cells were treated with 20 μM cisplatin and/or 20 μM #43 for 48 h and then harvested for Annexin V-FITC/PI double staining. Representative dot plots are shown in Figure 3C. Quantitative data revealed that apoptotic cells (including early and late apoptotic cells, Q3 + Q2 in the dot plots) induced by the combination treatment (46.70 ± 0.45%) was significantly increased compared to the DMSO control (16.23 ± 1.41%), cisplatin (27.09 ± 2.28%) or #43 alone (15.97 ± 2.58%), indicating that the combination of cisplatin and #43 synergistically induced apoptosis in MCF-7 cells (Figure 3D). Western blot analysis of apoptosis-related proteins was conducted 24 h after drug treatment. The combination of cisplatin and #43 markedly increased cleaved PARP while pro-apoptotic proteins, Bax and Bid were not affected (Figure 3E, left panel). The level of anti-apoptotic Bcl-2 was increased at 24 h, but significantly decreased after 48 h of combination treatment (Figure 3E, right panel). Quantitative data are illustrated in Figure 3F. Taken together, the combination of cisplatin and #43 induced apoptosis in MCF-7 cells.

### Induction of ROS by Cisplatin and #43

**FIGURE 4:**
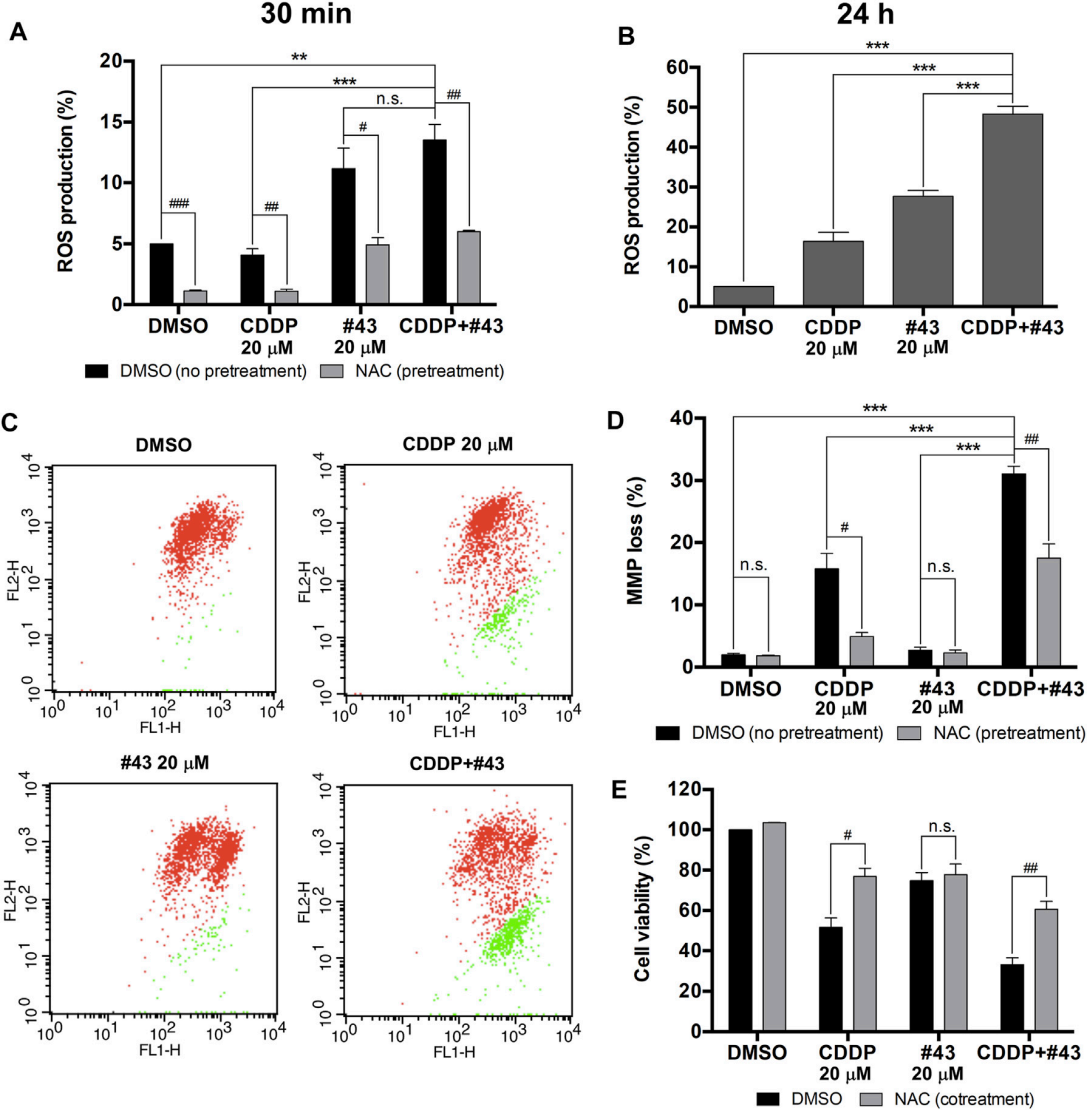
Induction of ROS and mitochondrial membrane potential loss by the combination of cisplatin and #43 in MCF-7 cells. **(A)** ROS production of cells treated with 20 μM cisplatin and/or 20 μM #43 for 30 min with or without 2 mM NAC pretreatment by the DCFH-DA assay. **(B)** ROS production of cells treated with 20 μM cisplatin and/or 20 μM #43 for 24 h. **(C)** Dot plots of JC-1 assay after 48 h of drug treatment to measure the mitochondrial membrane potential. Cells with intact MMP are labeled in red and cells with MMP loss are labeled in green. **(D)** Quantitative results of JC-1 flow cytometric analysis. NAC (2 mM) was added as an antioxidant which reversed MMP loss. **(E)** NAC rescued cells from growth inhibition by cisplatin and the combination of cisplatin and #43. Cells were treated with 20 μM cisplatin and/or 20 μM #43 for 72 h with or without 2 mM NAC, followed by the MTT assay. Data are presented as mean ± SEM of at least three independent experiments. *, *p* < 0.05; **, *p* < 0.01; ***, *p* < 0.001; n. s., not significant (*p* > 0.05) by one-way ANOVA followed by Bonferroni *t*-test. ^#^, *p* < 0.05; ^##^, *p* < 0.01; ^###^, *p* < 0.001; n. s., not significant (*p* > 0.05) by the two-tailed Student’s *t*-test.

Previous studies have revealed that both cisplatin and glucose starvation may increase ROS production (Choi et al., 2015; Ren and Shen, 2019), which is detrimental to cancer cells. Therefore, the combination effect of cisplatin and #43 on ROS production in MCF-7 cells was analyzed by the DCFH-DA assay. ROS production induced by the combination treatment (13.53 ± 1.28%) was not significantly different from that of #43 treatment alone (11.17 ± 1.69%) after a relatively short 30-min treatment, but there was a significant increase compared to the control (5.00 ± 0.02%) or cisplatin alone (4.08 ± 0.52%), indicating that #43 induced ROS more quickly than cisplatin alone. ROS production was markedly reversed by an antioxidant NAC (Figure 4A). ROS induction by the combination of cisplatin and #43 was more pronounced after 24 h of treatment (48.33 ± 1.97%) and significantly elevated compared to the control (5.03 ± 0.02%), cisplatin (16.37 ± 2.27%) or #43 alone (27.70 ± 1.42%) (Figure 4B).

### Induction of MMP Loss By Cisplatin Combined With #43

It has been reported that ROS are correlated with MMP to a certain extent (Marchi et al., 2012). Therefore, JC-1 assay was performed to determine the effect of cisplatin and #43 on mitochondrial integrity. A set of dot plots is shown in Figure 4C and quantitative results are illustrated in Figure 4D. The percentage of MCF-7 cells with MMP loss was markedly increased after combination treatment with cisplatin and #43 for 48 h (31.03 ± 1.23%) relative to the DMSO control (1.94 ± 0.23%) or #43 alone (2.67 ± 0.49%). Cells with MMP loss was also increased after cisplatin treatment (15.74 ± 2.46%), but to a much lesser extent. Furthermore, NAC also significantly reversed MMP loss induced by cisplatin or the combination of cisplatin and #43 (Figure 4D).

NAC was also used to verify whether ROS and MMP loss were involved in the growth inhibitory effect of the combination treatment. As shown in Figure 4E, NAC significantly reversed growth inhibition caused by cisplatin or the combination of cisplatin and #43, but not #43 alone, indicating that NAC was capable of attenuating the effect of cisplatin on cell growth.

Taken together, these results suggested that the combination of cisplatin and #43 may induce ROS generation and result in depolarization of mitochondrial membrane potential, leading to cell death in MCF-7 cells.

### #43 Enhances the DNA Damaging Effect of Cisplatin

**FIGURE 5:**
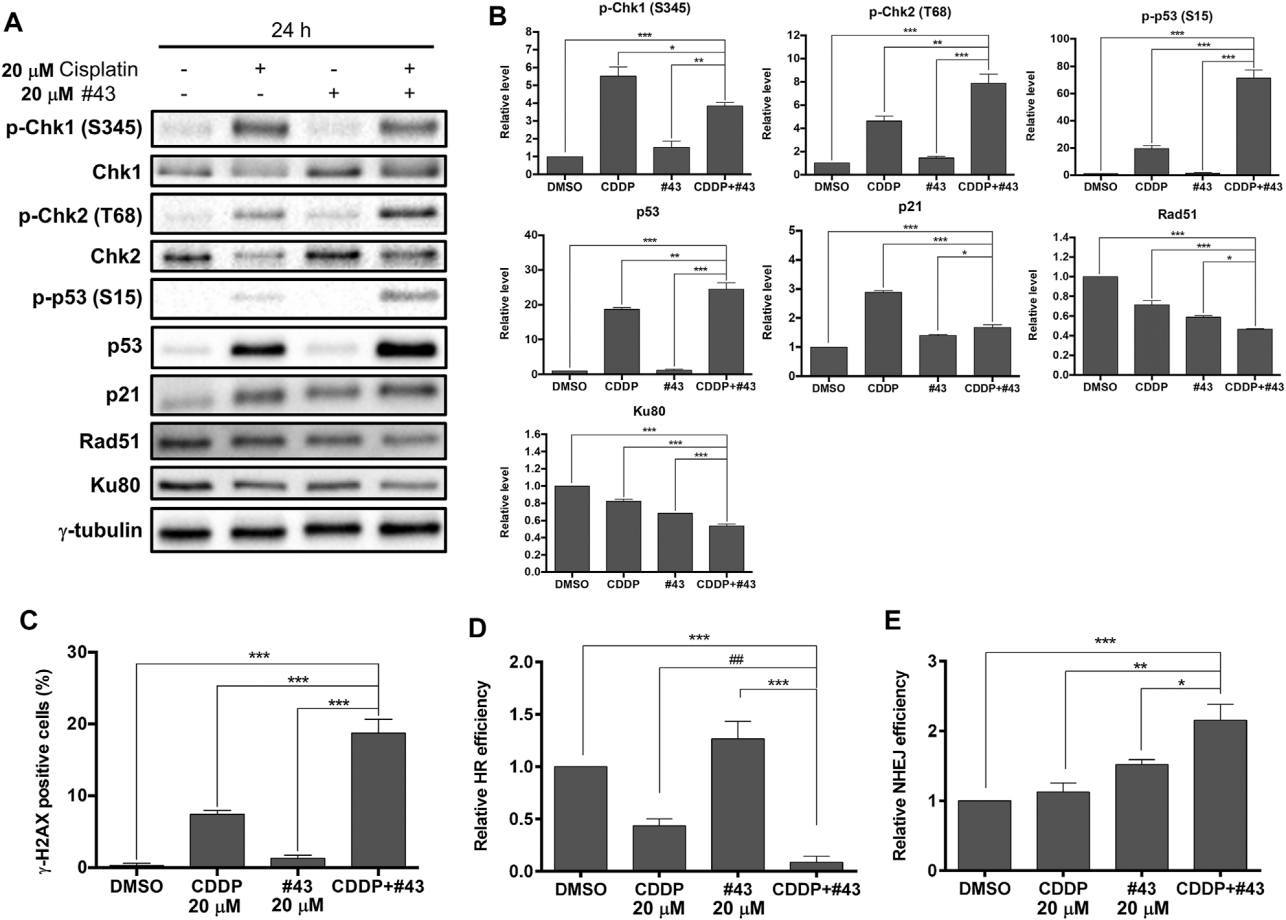
Effects of cisplatin and #43 on DNA damage response and DNA repair in MCF-7 cells. **(A)** Western blot analysis of DNA damage response and DNA repair-related proteins **(B)** Quantitative results of Western blot analysis. MCF-7 cells were treated with 20 μM cisplatin and/or 20 μM #43 for 24 h, and harvested for Western blot analysis. The relative protein levels were quantified by Image Lab software using γ-tubulin as the loading control. **(C)** Quantitative results of γ-H2AX immunofluorescence staining. MCF-7 cells were treated with 20 μM cisplatin and/or 20 μM #43 for 12 h, followed by immunofluorescence staining. Cells containing fluorescence intensity above the defined threshold were counted as γ-H2AX positive, and at least 100 cells were counted for each sample. **(D)** Quantitative results of HR assay. **(E)** Quantitative results of NHEJ assay. Cells for HR and NHEJ assays were treated with 20 μM cisplatin and/or 20 μM #43 for 24 h. Data are presented as mean ± SEM of at least three independent experiments. *, *p* < 0.05; **, *p* < 0.01; ***, *p* < 0.001 by one-way ANOVA followed by Bonferroni *t*-test. ^##^, *p* < 0.01 by the two-tailed Student’s *t*-test.

Cisplatin can cause DNA damage through the formation of intrastrand or interstrand crosslinks with DNA, and the cisplatin-DNA adducts prevent cancer cells from DNA replication and cell cycle progression, leading to cell death via apoptosis (Dasari and Tchounwou, 2014). To determine whether #43 affected DNA damage induced by cisplatin, Western blot analysis of proteins involved in the DDR and its downstream signaling pathways was performed. A set of results is shown in Figure 5A and quantitative results are illustrated in Figure 5B. The phosphorylation of two checkpoint kinases, Chk1 on serine 345 and Chk2 on threonine 68, was dramatically increased after MCF-7 cells were treated with cisplatin for 24 h, but #43 only slightly increased p-Chk1 (S345) and p-Chk2 (T68) (1.52-fold and 1.43-fold for Chk1 and Chk2, respectively relative to the control group). The combination with #43 significantly enhanced the phosphorylation of Chk2 (7.88-fold of the control) compared to cisplatin alone (4.63-fold). Interestingly, the combination with #43 decreased the p-Chk1 (S345) level relative to cisplatin alone, which was still significantly higher than the untreated control. Phosphorylation and expression of p53, the downstream effector of the DDR, were significantly increased by the combination treatment compared to cisplatin alone, while #43 alone did not show any obvious effect on p53. Although cisplatin also induced p21, a major downstream mediator of p53-dependent cell cycle arrest (Bohgaki et al.), the combination of cisplatin with #43 downregulated p21 induced by cisplatin (Figures 5A,B), which may be an indicator of a switch to apoptosis. Induction of DNA damage was evaluated by immunofluorescence staining of γ-H2AX. As shown in Figure 5C, 20 μM cisplatin alone increased the percentage of γ-H2AX positive cells (7.43 ± 0.95%) compared to the DMSO control (0.29 ± 0.29%) or 20 μM #43 alone (1.27 ± 0.45%), and the combination treatment further enhanced the effect of cisplatin up to 18.70 ± 1.97%. These results suggested that cisplatin activated DDR and induced DNA damage, and #43 may further potentiate the DNA damaging effect of cisplatin.

Proteins involved in the DNA repair systems were also analyzed. Both Rad51 and Ku80 required for DNA repair *via* HR and NHEJ, respectively, were downregulated in MCF-7 cells treated with the combination of cisplatin and #43 for 24 h (Figures 5A,B). HR assays further confirmed that the combination of cisplatin and #43 compromised DNA repair *via* HR (Figure 5D). Cisplatin alone downregulated Rad51 and suppressed HR, while the combination with #43 further enhanced this effect with significantly lower Rad51 levels (*p* < 0.001 by one-way ANOVA vs. cisplatin, Figure 5B) and relative HR efficiency (*p* = 0.065 by one-way ANOVA, *p* = 0.007 by two-tailed Student’s *t*-test vs. cisplatin, Figure 5D). Although #43 alone also downregulated Rad51, it slightly enhanced HR efficiency relative to the DMSO control (Figures 5A,B,D). NHEJ assays revealed that the combination of cisplatin and #43 increased NHEJ (Figure 5E) in spite of downregulation of Ku80 (Figures 5A,B), which may lead to accumulation of more DNA damage as illustrated in Figure 5C. Taken together, these results indicated that the combination with #43 may enhance the cytotoxicity of cisplatin through increasing its DNA damaging effect and undermining the error-free DNA repair via HR while increasing error-prone DNA repair *via* NHEJ.

### Effects of Cisplatin and #43 on the Akt/mTOR Signaling Pathway

**FIGURE 6:**
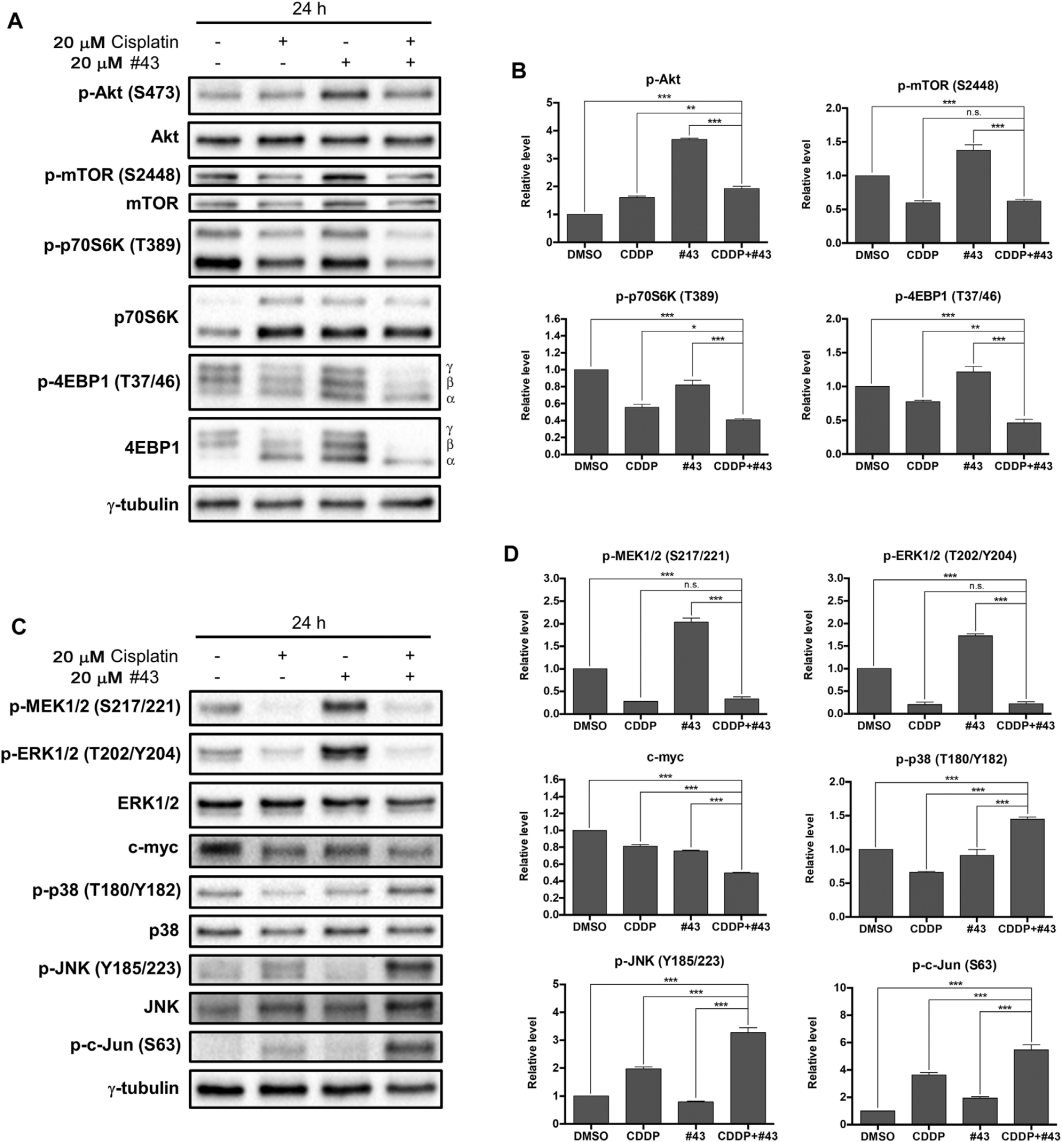
Effects of cisplatin and #43 on proteins involved in the Akt/mTOR and MAPK signaling pathways in MCF-7 cells. **(A)** Western blot analysis of the Akt/mTOR signaling pathway-related proteins. The α, β, γ isoforms represent the phosphorylation status of 4EBP1. γ-4EBP1 is the hyperphosphorylated isofrom and α-4EBP1 is hypophosphorylated isoform. **(B)** Quantitative results of Western blot analysis of proteins related to the Akt/mTOR signaling pathway. **(C)** Western blot analysis of the MAPK signaling pathway-related proteins. **(D)** Quantitative results of Western blot analysis of proteins related to the MAPK signaling pathway. Cells were treated with 20 μM cisplatin and/or 20 μM #43 for 24 h, and harvested for Western blot analysis. The relative protein levels were quantified by Image Lab software using γ-tubulin as the loading control. Data are presented as mean ± SEM of at least three independent experiments. *, *p* < 0.05; **p < 0.01; ***, *p* < 0.001; n. s., not significant (*p* > 0.05) by one-way ANOVA followed by Bonferroni *t*-test.

The Akt/mTOR signaling pathway is frequently overactivated in various types of cancer which may promote malignant cell survival and cancer progression (Porta et al., 2014). Therefore, whether the Akt/mTOR signaling pathway was involved in the synergistic cytotoxicity of cisplatin and #43 was investigated. Surprisingly, phosphorylation of Akt on serine 473 (p-Akt) was not only slightly increased by cisplatin, but also markedly induced by #43 which was suppressed by combination with cisplatin (Figures 6A,B). Interestingly, phosphorylation of mTOR, a downstream signaling protein of Akt, was decreased by both cisplatin and the combination treatment, but significantly increased after the #43 single treatment. Furthermore, phosphorylation of the downstream effector proteins, p70S6K and 4EBP1, was also significantly diminished after the combination treatment compared to single treatments. Not only the total phosphorylation level of 4EBP1 was decreased, but there was also a shift from hyperphosphorylated γ-4EBP1 to hypophosphorylated α-4EBP1. However, #43 alone only slightly decreased p-p70S6K (T389) and increased p-4EBP1 (T37/46) levels compared to the DMSO control (Figures 6A,B). These data indicated that the combination of cisplatin and #43 inhibited the Akt/mTOR downstream signaling pathway but not Akt phosphorylation, suggesting that Akt downstream signaling proteins can be regulated by proteins other than Akt. Nevertheless, the Akt/mTOR signaling pathway may be partially involved in the synergistic mechanism of cisplatin and #43 since cisplatin suppressed the induction of p-Akt, p-mTOR and p-4EBP1 by #43 treatment.

### Effects of Cisplatin and #43 on the MAPK Signaling Pathway

The MAPK signaling pathway regulates diverse physiological functions including cell proliferation, differentiation, development, inflammation, and apoptosis (Morrison, 2012). To determine whether the MAPK signaling pathway was related to the synergism between cisplatin and #43, expression and phosphorylation of three main MAPK proteins, extracellular signal-regulated kinase (ERK), c-Jun NH2-terminal kinase (JNK), and p38, as well as some upstream and downstream signaling proteins were analyzed. Strikingly, #43 alone markedly increased the level of p-ERK1/2 (T202/204). Similarly, the level of p-MEK (S217/221), which is upstream of ERK, was also increased by #43, indicating that changes in ERK1/2 phosphorylation may be due to the effect of #43 on MEK activation. However, phosphorylation of MEK and ERK induced by #43 was dramatically downregulated by combination with cisplatin. The level of c-myc, a downstream transcription factor of MEK/ERK, was also significantly decreased after the combination treatment, suggesting that the combination of cisplatin and #43 may hinder cell proliferation through inactivation of the ERK signaling pathway (Figures 6C,D).

On the other hand, cisplatin increased phosphorylation of JNK, and its downstream effector protein, c-Jun, and the combination with #43 further enhanced this effect. #43 alone did not significantly affect phosphorylation of either JNK or c-Jun relative to the DMSO control. Similar to JNK, p38 was activated after the combination treatment, but there was no significant change caused by #43, and p-p38 was even downregulated by cisplatin. Thus, activation of JNK and p38 may also contribute to the synergism of the combination treatment (Figures 6C,D).

### Effects of Akt and the ERK Signaling Pathway on Growth Inhibition by #43

**FIGURE 7:**
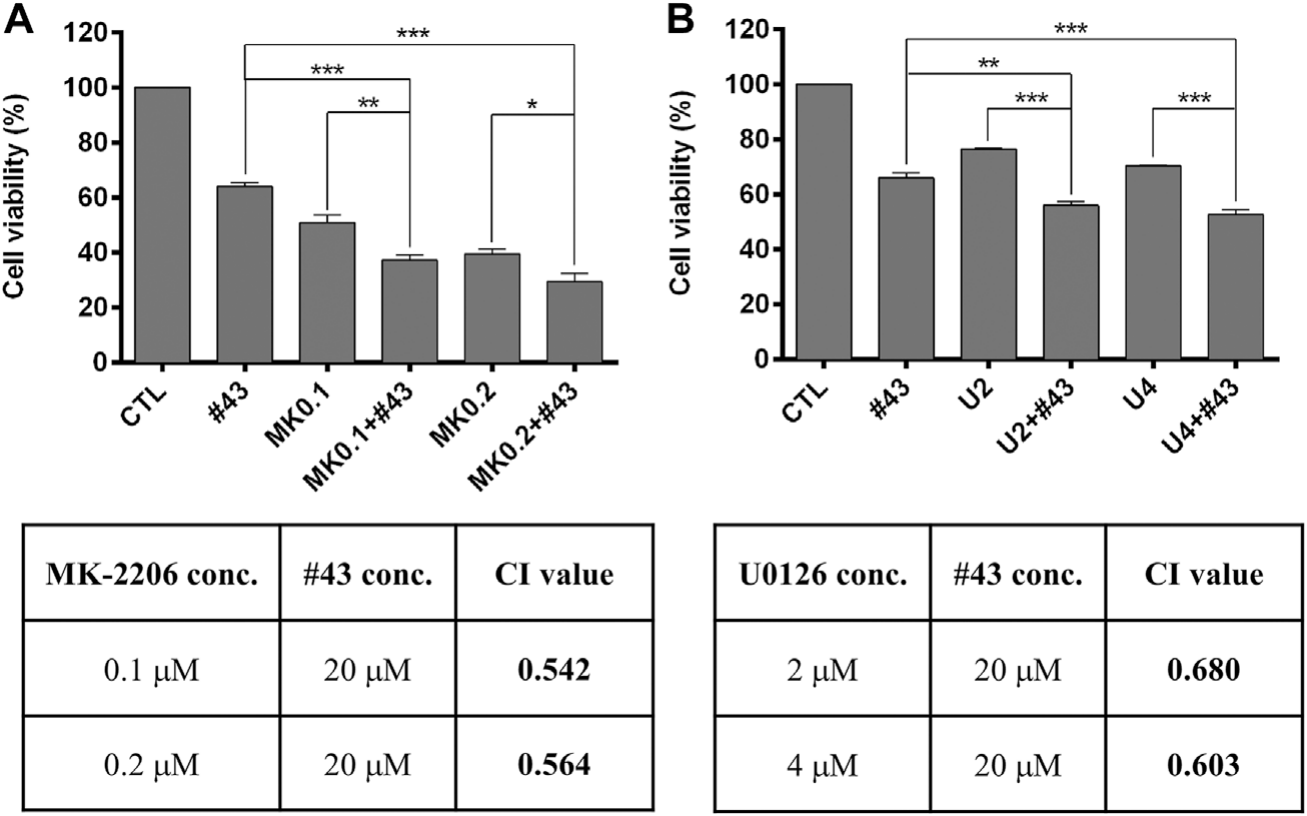
MK-2206 and U0126 potentiate the growth inhibitory effect of #43 in MCF-7 cells. **(A)** Viability of cells treated with 20 μM #43 either alone or in combination with 0.1 and 0.2 μM MK-2206 (MK0.1, MK0.2) for 72 h, followed by MTT assay. **(B)** Viability of cells treated with 20 μM #43 either alone or in combination with 2 and 4 μM U0126 (U2, U4) for 72 h, followed by MTT assay. The combination index (CI) was calculated by Compusyn software. Data are presented as mean ± SEM of at least three independent experiments. *, *p* < 0.05; **p < 0.01; ***, *p* < 0.001 by one-way ANOVA followed by Bonferroni *t*-test.

Since Akt and the ERK signaling pathway were activated by #43 in MCF-7 cells (Figure 6), an Akt inhibitor MK-2206 and a MEK inhibitor U0126 were combined with #43 to determine their effects on growth inhibition by #43. Results from the MTT assay showed that both MK-2206 and U0126 potentiated the growth inhibitory effect of #43. The combination of 0.1 or 0.2 μM MK-2206 with 20 μM #43 showed synergism with CI values of 0.542 and 0.564, respectively, (Figure 7A). The combination of 2 or 4 μM U0126 with 20 μM #43 also showed synergism with CI values of 0.680 and 0.603, respectively, (Figure 7B). Similar results were obtained when 20 μM #43 was combined with 1 or 2 μM MK-2206, and 5 or 10 μM U0126 in MDA-MB-231 cells (Supplementary Figure S5). Thus, activation of Akt and ERK signaling pathway caused by #43 may play a protective role not only in MCF-7 but also in MDA-MB-231 cells, which may attenuate the growth inhibitory effect of #43.

## Discussion

It has been reported that high expression of GLUT1 is associated with unfavorable overall survival and disease-free survival rates in various tumors. Moreover, overexpression of GLUT1 is also significantly associated with poor differentiated tumors, positive lymph node metastasis, and larger tumor size according to a meta-analysis (Yu et al., 2017). GLUT1 inhibitors such as WZB117 have been reported to sensitize or increase efficacy of traditional therapeutics including adriamycin (doxorubicin) and radiotherapy for breast cancer (Chen et al., 2017; Zhao et al., 2016). In this study, we found that #43, a novel GLUT1 inhibitor identified in our laboratory, synergized with cisplatin, a commonly used chemotherpeutic drug, not only in ER (+) MCF-7 cells but also in triple-negative MDA-MB-231 breast cancer cells which are usually more resistant to drug treatment (Figure 1). The combination of cisplatin and #43 also displayed a moderate synergistic effect in another ER (+) breast cancer cell line T47D (Supplementary Figure S6). Interestingly, other GLUT1 inhibitors, such as BAY-876, only showed an additive effect with cisplatin in MCF-7 cells (Supplementary Figure S7). The IC_50_ values of cisplatin and #43 were estimated to be 10 and 60 μM, respectively, in single treatments, and in combination treatment the IC_50_ was 5.7 μM in MCF-7 cells. The combination of 20 μM of cisplatin and 20 μM of #43 was used for most of our mechanism studies. According to a report by Rajkumar et al. (Rajkumar et al., 2016), the Cmax of total cisplatin was 5.37 ± 1.47 μg/ml at the end of 1 h infusion, which was about 20 μM of cisplatin, supporting the *in vivo* relevance. Whether the concentration of #43 can reach 20 μM *in vivo* remains to be determined in future studies. Since #43 was less potent, it could be reasonable and safe to use the concentration range tested in this study to enhance the anticancer activity of cisplatin. Thus, this combination strategy may be beneficial and could prevent serious side effects caused by high-dose cisplatin.

Cisplatin has been reported to induce S and G2/M cell cycle arrest in leukemia cells (Velma et al., 2016), and WZB117 caused G0/G1 cell cycle arrest in NSCLC cells (Liu et al., 2012). Mechanism study in MCF-7 revealed that cisplatin induced S and G2/M arrest and #43 caused G0/G1 arrest. The combination of cisplatin and #43 did not arrest the cell cycle as obvious as single agents, but significantly increased the subG1 population, indicative of induction of apoptosis which was confirmed by the Annexin V-FITC/PI apoptosis assay (Figures 3B,C). Thus, the combination of cisplatin and #43 displayed mixed effects of cisplatin and #43 on cell cycle progression and ultimately led to apoptosis.

Excessive ROS accumulation may be detrimental to cells, causing imbalance of mitochondrial redox status and impairment of biomolecules. It has been reported that cisplatin is capable of inducing ROS in mitochondria in A549 and DU145 cells treated with 10 and 20 μM cisplatin, respectively, for 16 h (Marullo et al., 2013). Cisplatin-induced ROS generation can also cause mitochondrial damage and inhibit both glycolysis and the TCA cycle, which are crucial pathways for energy production (Choi et al., 2015). Inhibition of GLUT1 was previously demonstrated to elevate ROS levels in muscle cells (Andrisse et al., 2014). We found that 20 μM #43 alone and the combination of 20 μM cisplatin and 20 μM #43, but not 20 μM cisplatin, significantly induced ROS within 30 min which was reversed by NAC in MCF-7 cells. ROS induction by the combination treatment was further elevated at 24 h (Figures 4A,B). It has been reported that ROS may cause oxidation of adenine nucleotide translocator, resulting in the opening of mitochondrial permeability transition pore (mPTP) and the collapse of MMP (Marchi et al., 2012). We found that cisplatin induced MMP loss, and the combination treatment increased MMP loss to a greater extent. Although #43 induced ROS generation in MCF-7 cells, it did not cause MMP loss (Figure 4). It has been demonstrated that p53 opens mPTP leading to the collapse of MMP (Vaseva et al., 2012). We found that cisplatin alone and the combination of cisplatin and #43 activated p53 (Figures 5A,B), suggesting that p53 may play a role in drug-induced MMP loss. Furthermore, NAC not only reversed ROS production and MMP loss, but also rescued cells from growth inhibition caused by cisplatin and #43 (Figure 4E). Taken together, these results suggested that the combination of cisplatin and #43 significantly induced ROS generation, and led to the collapse of MMP, which may contribute to growth inhibition and apoptosis.

Cisplatin is a chemotherapeutic agent that forms crosslinks with DNA molecules, leading to DNA damage and activation of DDR (Florea and Büsselberg, 2011; Dasari and Tchounwou, 2014). Glucose is an important factor for redox homeostasis and chromatin remodeling, and deregulation of glucose affects DNA organization, DNA repair, and even promotes DNA damage (Turgeon et al., 2018). Western blot analysis showed that the combination of cisplatin and #43 may trigger DDR mainly *via* activating Chk2, a crucial checkpoint kinase (Bohgaki et al., 2010). #43 may potentiate cisplatin-induced DSBs, which is consistent with the increase in γ-H2AX positive cells (Figure 5). The p53 tumor suppressor protein was greatly induced and activated when MCF-7 cells were treated with the combination of cisplatin and #43, which may act in concert with the induction of the apoptotic pathway. Furthermore, Rad51 involved in error-free HR was downregulated (Figures 5A,B) and HR efficiency was compromised by the combination of cisplatin and #43 (Figure 5D). Although Ku80 was downregulated (Figures 5A,B), error-prone NHEJ was increased by the combination of cisplatin and #43 (Figure 5E), which may in part contribute to DNA damage induction. These results suggested that although #43 alone did not lead to clear activation of DDR, it could further reinforce the DNA damaging effect and also impair the DNA repair system when combined with cisplatin, leading to cell death.

The Akt/mTOR signaling pathway plays a vital role in cancer survival and progression (Porta et al., 2014). It has been reported that cisplatin induces Akt activation mediated by EGFR, leading to cisplatin resistance (Florea and Büsselberg, 2011; Dasari and Tchounwou, 2014). WZB117, a GLUT1 inhibitor has been demonstrated to reduce Akt phosphorylation in NSCLC cells (Liu et al., 2012) or have no significant effect on p-Akt in MCF-7 or MDA-MB-231 cells (Li et al., 2019). Here we found that the p-Akt level was only slightly increased by cisplatin, but was significantly elevated by #43 treatment in MCF-7 cells (Figures 6A,B), which was associated with cell survival since Akt inhibitor MK-2206 enhanced the growth inhibitory effect of #43 (Figure 7A). It has been reported that glucose deprivation activates Akt to protect cells from death (Gao et al., 2014). Thus, the lack of glucose resulting from inhibition of GLUT1 by #43 may increase cellular stresses, leading to Akt activation. When combined with cisplatin, p-Akt induced by #43 was greatly suppressed and the level fell in between that induced by either cisplatin or #43 alone (Figures 6A,B). Akt is an upstream positive regulator of mTOR. However, mTOR can also be regulated through various pathways, such as the MAPK pathway or other pathways that signal the availability of nutrients (Memmott and Dennis, 2009). Cisplatin suppressed but #43 induced mTOR phosphorylation (Figures 6A,B). The combination treatment also inhibited mTOR activation by #43, and the phosphorylation of p70S6K and 4EBP1, downstream effectors of Akt/mTOR, was significantly downregulated compared to single treatments. Taken together, these data suggested that the combination of cisplatin and #43 may hinder cell growth through inhibiting the mTOR downstream signaling pathway(s) independent of Akt.

Previous studies have shown that the MAPK pathway is involved in cisplatin-induced cell death. Cisplatin induced ERK, JNK, and p38 activation, leading to cell apoptosis *via* p53 activation (Florea and Büsselberg, 2011; Dasari and Tchounwou, 2014). Similarly, a GLUT inhibitor phloretin has been demonstrated to increase phosphorylation of JNK, p38, and ERK in breast tumor cells and NSCLC cells (Kim et al., 2009; Min et al., 2015). However, GLUT1 inhibition was also shown to decrease the levels of phosphorylated JNK and c-Jun (Koch et al., 2015). Our Western blotting data showed that ERK signaling was upregulated by #43 *via* MEK activation, and both cisplatin alone and the combination of cisplatin and #43 significantly inhibited ERK activation. Moreover, c-myc expression level was reduced after combination treatment compared to either single agent. Although the role of the ERK signaling pathway in apoptosis remains controversial, our data suggested that upregulation of ERK signaling pathway by #43 was prosurvival since the MEK inhibitor U0126 increased the growth inhibitory effect of #43 (Figure 7B). The combination treatment activated both p38, JNK and its downstream transcription factor c-Jun in MCF-7 cells (Figures 6C,D). It has been reported that the activation of p38 and the JNK signaling pathway induces apoptosis (Cai et al., 2006; Dhanasekaran and Reddy, 2017). Thus, cisplatin combined with #43 may show synergistic cytotoxic effect via downregulation of the ERK signaling pathway and upregulation of both p38 and the JNK signaling pathway.

## Conclusion

**FIGURE 8:**
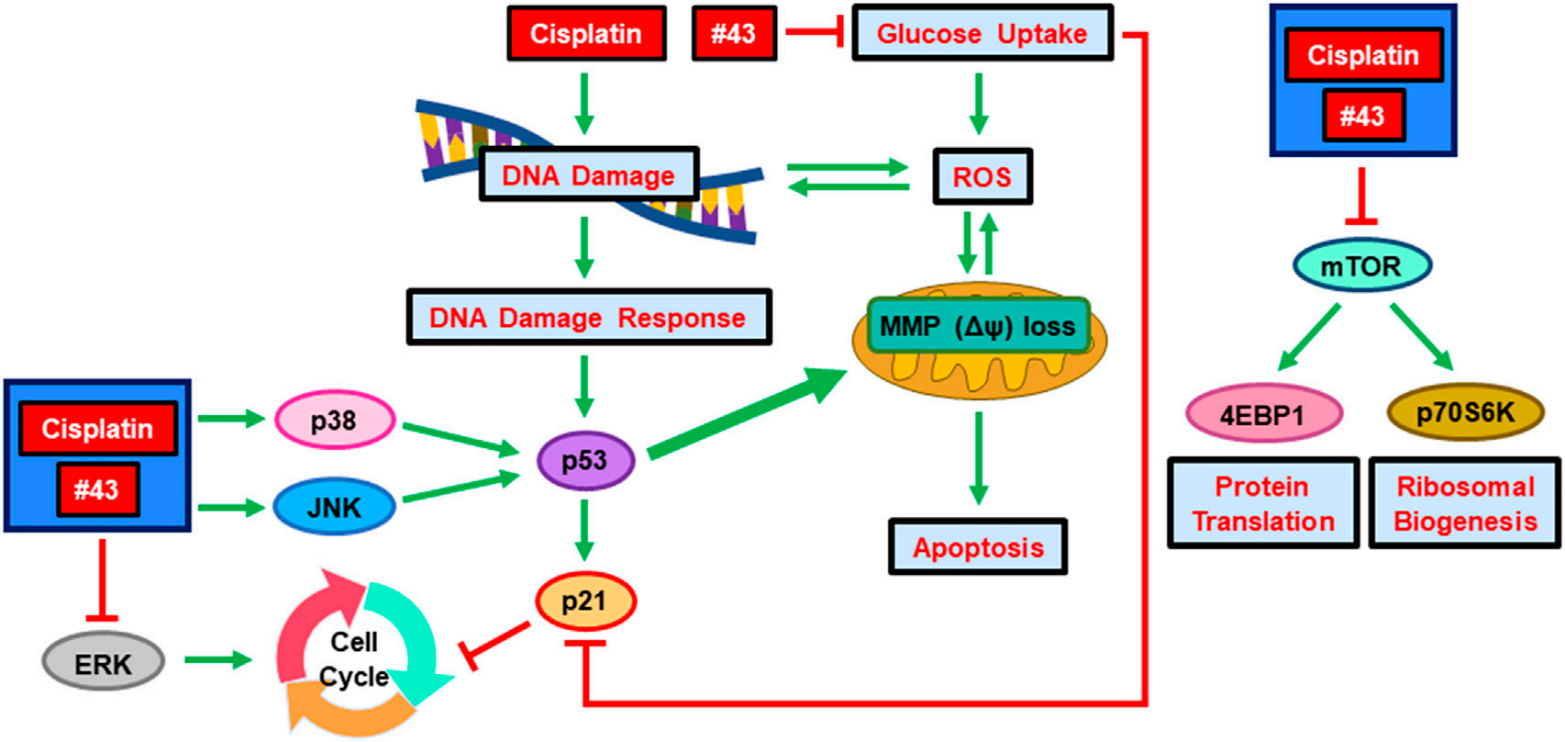
Schematic diagram of the potential molecular mechanisms underlying the synergism of cisplatin and a novel GLUT1 inhibitor #43. Green arrows indicate induction and red lines denote inhibition.

The combination of cisplatin and #43 exerted a synergistic cytotoxic effect in both ER (+) and triple-negative breast cancer cells. The potential underlying mechanisms are illustrated in Figure 8. Cisplatin and #43 caused cell cycle arrest at S + G2/M and G0/G1, respectively. Moreover, #43 potentiated cisplatin-induced DDR and also impeded the DNA repair systems. Elevation of ROS levels and the impairment of mitochondrial membrane potential may ultimately lead to apoptosis. Furthermore, inactivation of the Akt/mTOR and ERK signaling pathways and activation of p38 and the JNK signaling pathway may also contribute to cell death induced by the combination of cisplatin and #43. We demonstrated that Akt inhibitor MK-2206 and MEK inhibitor U0126 also synergized with #43, providing a rationale for the combination of #43 with Akt/mTOR or MEK/ERK inhibitors in cancer treatment. Furthermore, *GLUT1* knockdown in combination with cisplatin displayed a similar synergistic growth inhibitory effect to that of the #43 and cisplatin combination, suggesting that the effect of #43 is associated with GLUT1 inhibition. However, we cannot exclude the possibility that other yet to be identified off-target effects of #43 may also play a role. Furthermore, it remains to be determined whether #43 can also inhibit other GLUT isoforms. *In vivo* studies will be conducted in the future to provide supporting evidence for potential clinical applications.

## Data Availability

The original contributions presented in the study are included in the article/Supplementary Material, further inquiries can be directed to the corresponding authors.
