# Supplementary material for: The Combination of a Novel GLUT1 Inhibitor and Cisplatin Synergistically Inhibits Breast Cancer Cell Growth By Enhancing the DNA Damaging Effect and Modulating the Akt/mTOR and MAPK Signaling Pathways
Source: Front Pharmacol. 2022 May 19;13:879748. doi: 10.3389/fphar.2022.879748 (PMC9160228; doi:10.3389/fphar.2022.879748)
Supplement: Supplementary file 1 [file Presentation1.PPT]

## Slide 1
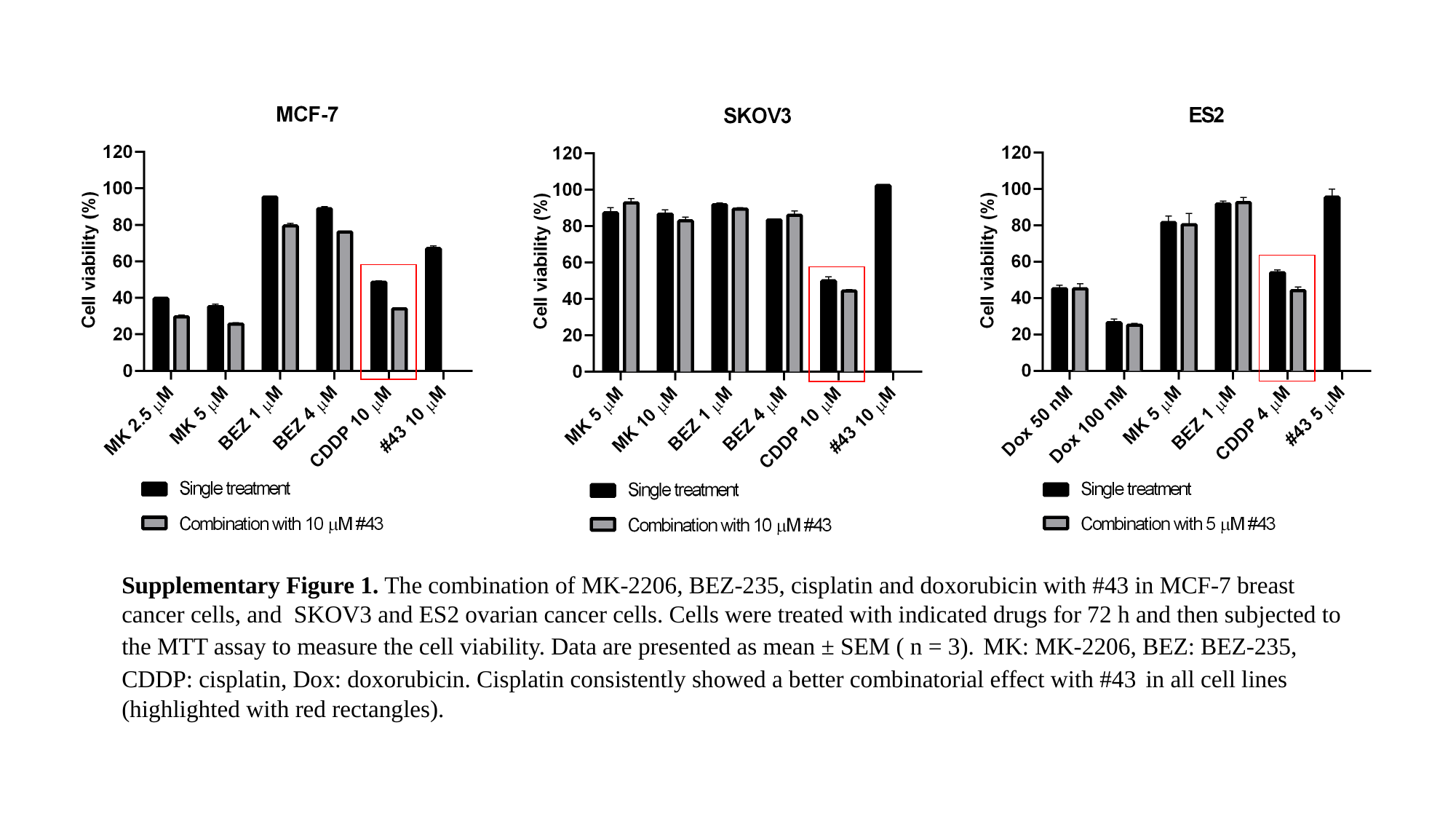

Supplementary Figure 1. The combination of MK-2206, BEZ-235, cisplatin and doxorubicin with #43 in MCF-7 breast cancer cells, and SKOV3 and ES2 ovarian cancer cells. Cells were treated with indicated drugs for 72 h and then subjected to the MTT assay to measure the cell viability. Data are presented as mean ± SEM ( n = 3). MK: MK-2206, BEZ: BEZ-235, CDDP: cisplatin, Dox: doxorubicin. Cisplatin consistently showed a better combinatorial effect with #43 in all cell lines (highlighted with red rectangles).

## Slide 2
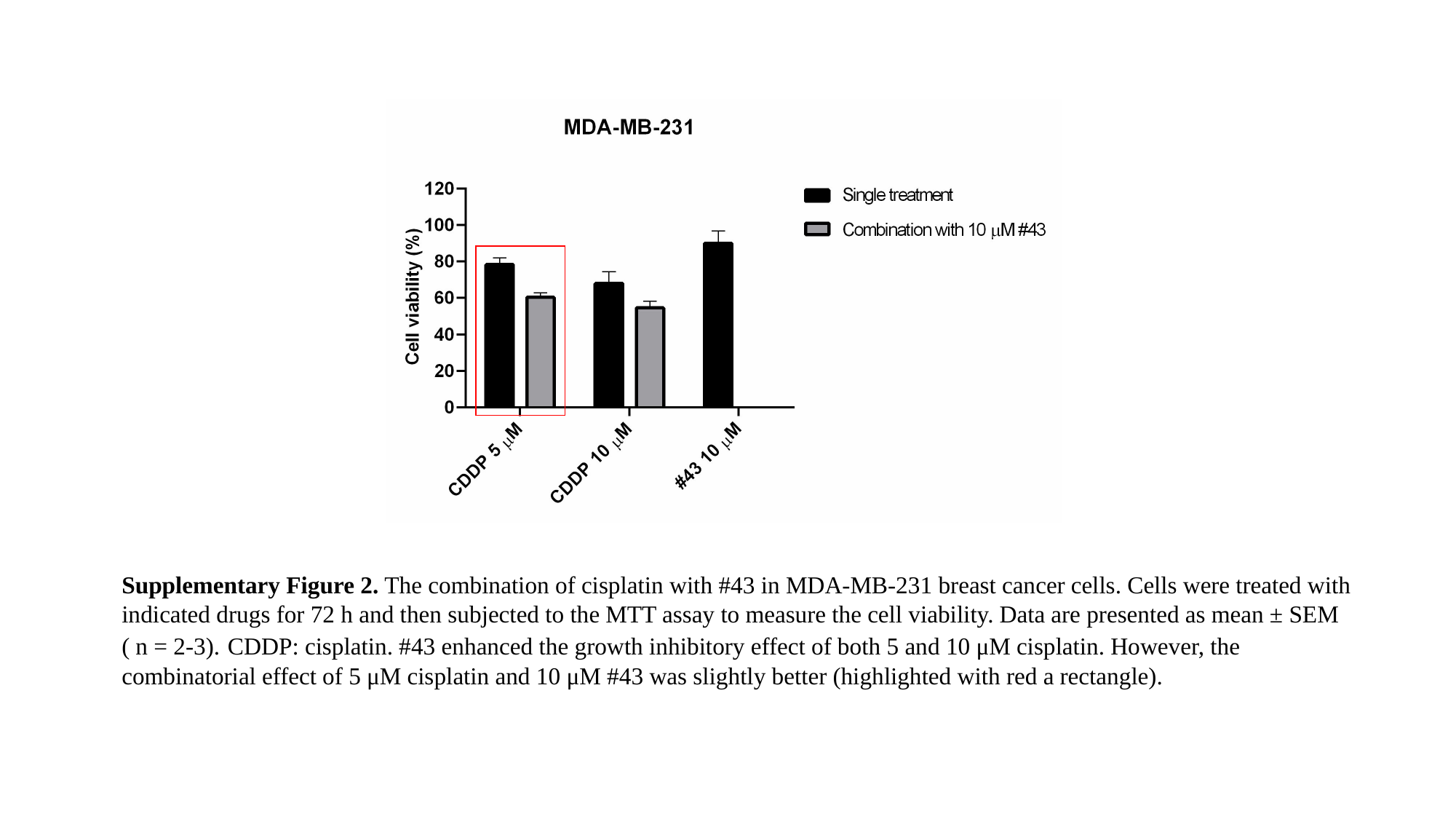

Supplementary Figure 2. The combination of cisplatin with #43 in MDA-MB-231 breast cancer cells. Cells were treated with indicated drugs for 72 h and then subjected to the MTT assay to measure the cell viability. Data are presented as mean ± SEM ( n = 2-3). CDDP: cisplatin. #43 enhanced the growth inhibitory effect of both 5 and 10 μM cisplatin. However, the combinatorial effect of 5 μM cisplatin and 10 μM #43 was slightly better (highlighted with red a rectangle).

## Slide 3
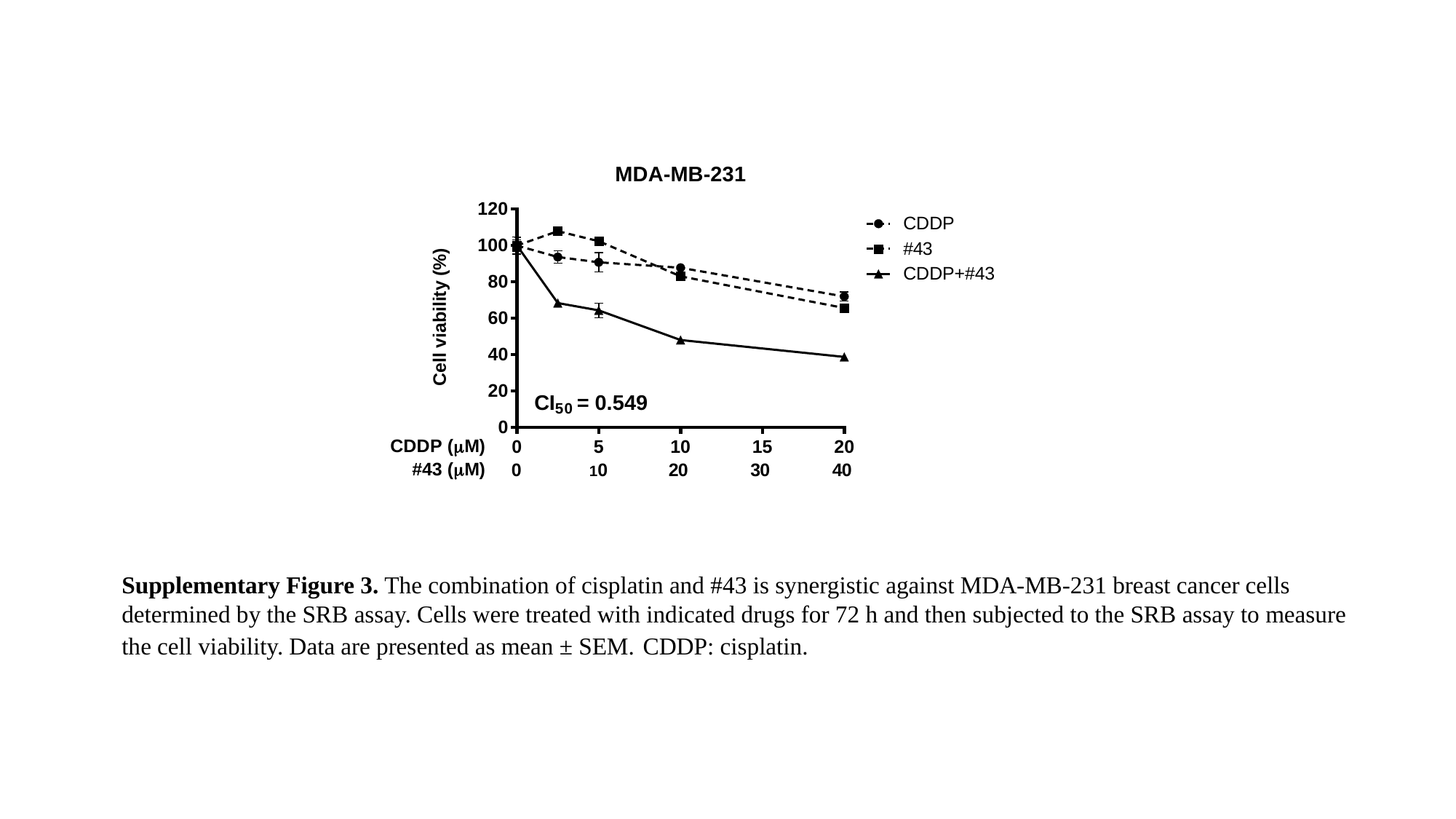

Supplementary Figure 3. The combination of cisplatin and #43 is synergistic against MDA-MB-231 breast cancer cells determined by the SRB assay. Cells were treated with indicated drugs for 72 h and then subjected to the SRB assay to measure the cell viability. Data are presented as mean ± SEM. CDDP: cisplatin.

## Slide 4
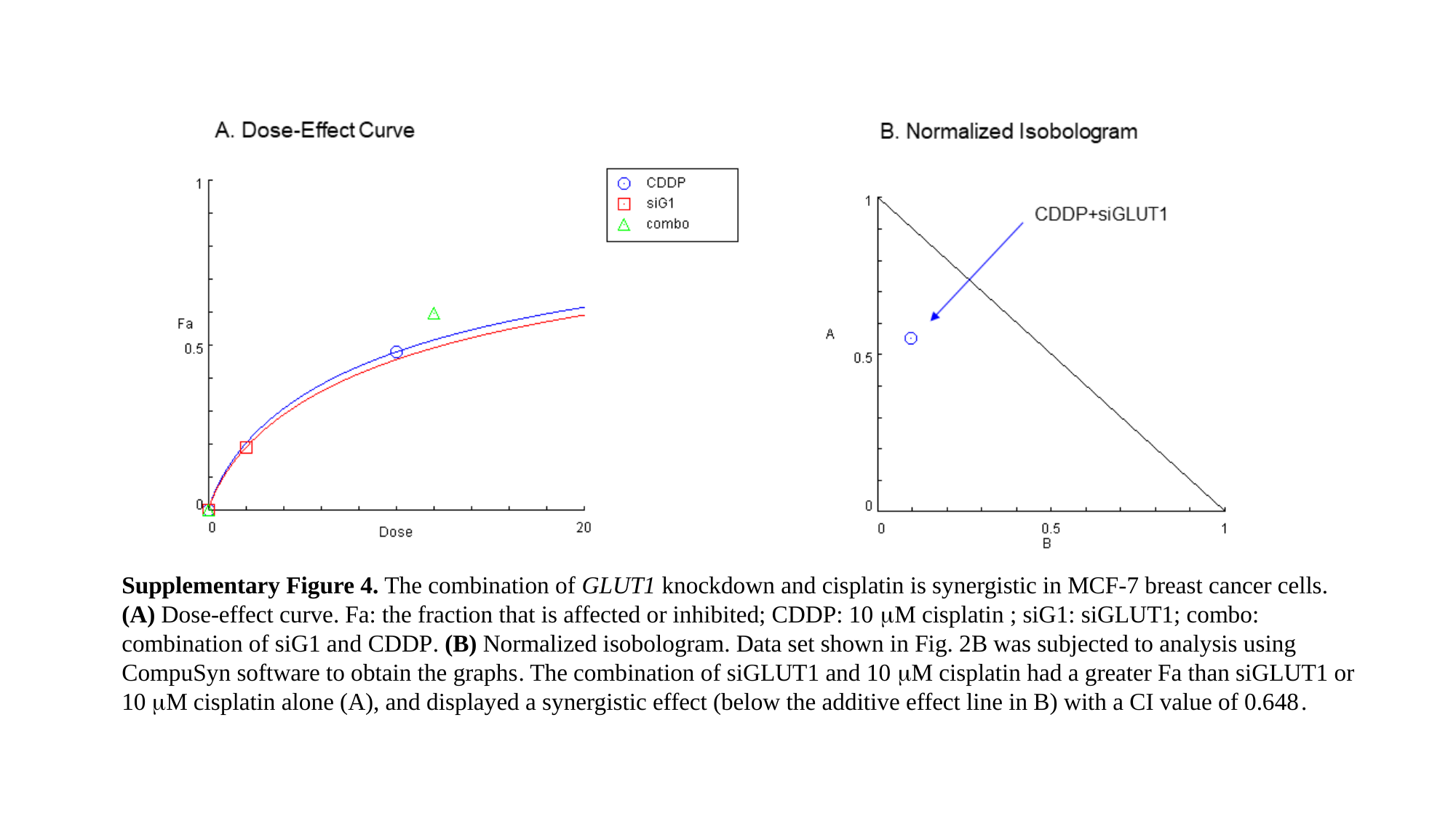

Supplementary Figure 4. The combination of GLUT1 knockdown and cisplatin is synergistic in MCF-7 breast cancer cells. (A) Dose-effect curve. Fa: the fraction that is affected or inhibited; CDDP: 10 μM cisplatin ; siG1: siGLUT1; combo: combination of siG1 and CDDP. (B) Normalized isobologram. Data set shown in Fig. 2B was subjected to analysis using CompuSyn software to obtain the graphs. The combination of siGLUT1 and 10 μM cisplatin had a greater Fa than siGLUT1 or 10 μM cisplatin alone (A), and displayed a synergistic effect (below the additive effect line in B) with a CI value of 0.648.

## Slide 5
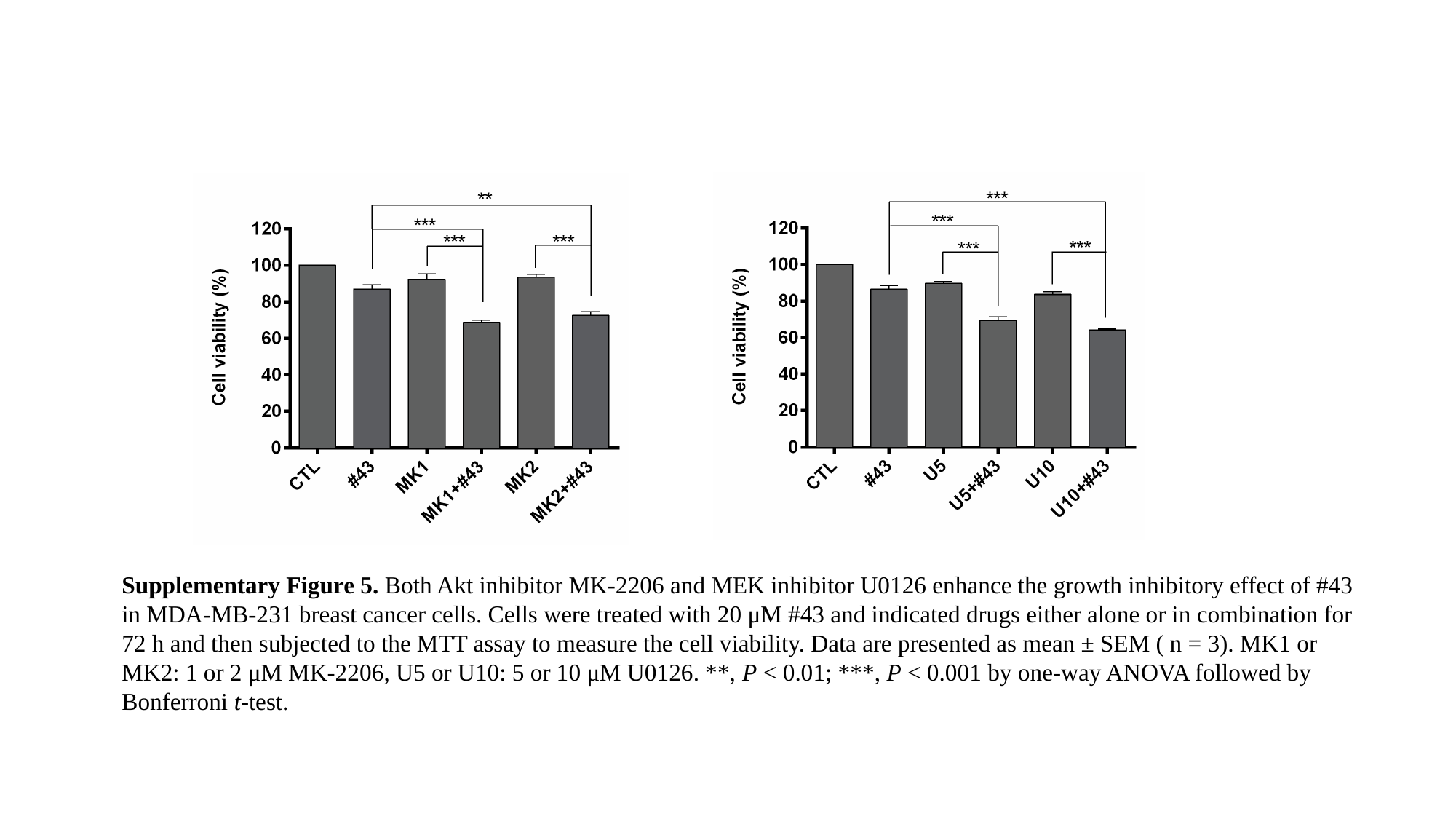

Supplementary Figure 5. Both Akt inhibitor MK-2206 and MEK inhibitor U0126 enhance the growth inhibitory effect of #43 in MDA-MB-231 breast cancer cells. Cells were treated with 20 μM #43 and indicated drugs either alone or in combination for 72 h and then subjected to the MTT assay to measure the cell viability. Data are presented as mean ± SEM ( n = 3). MK1 or MK2: 1 or 2 μM MK-2206, U5 or U10: 5 or 10 μM U0126. **, P < 0.01; ***, P < 0.001 by one-way ANOVA followed by Bonferroni t-test.

## Slide 6
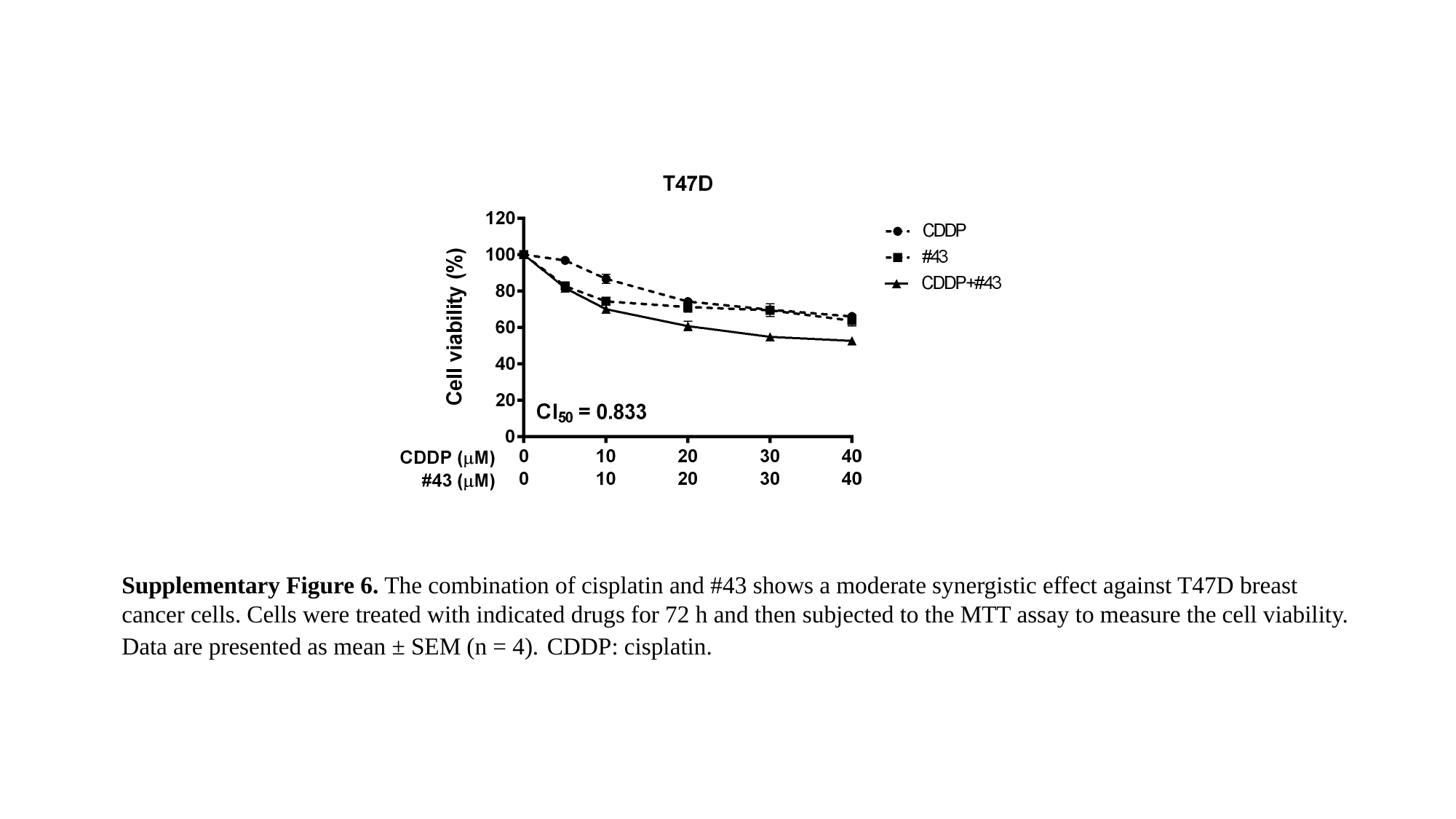

Supplementary Figure 6. The combination of cisplatin and #43 shows a moderate synergistic effect against T47D breast cancer cells. Cells were treated with indicated drugs for 72 h and then subjected to the MTT assay to measure the cell viability. Data are presented as mean ± SEM (n = 4). CDDP: cisplatin.

## Slide 7
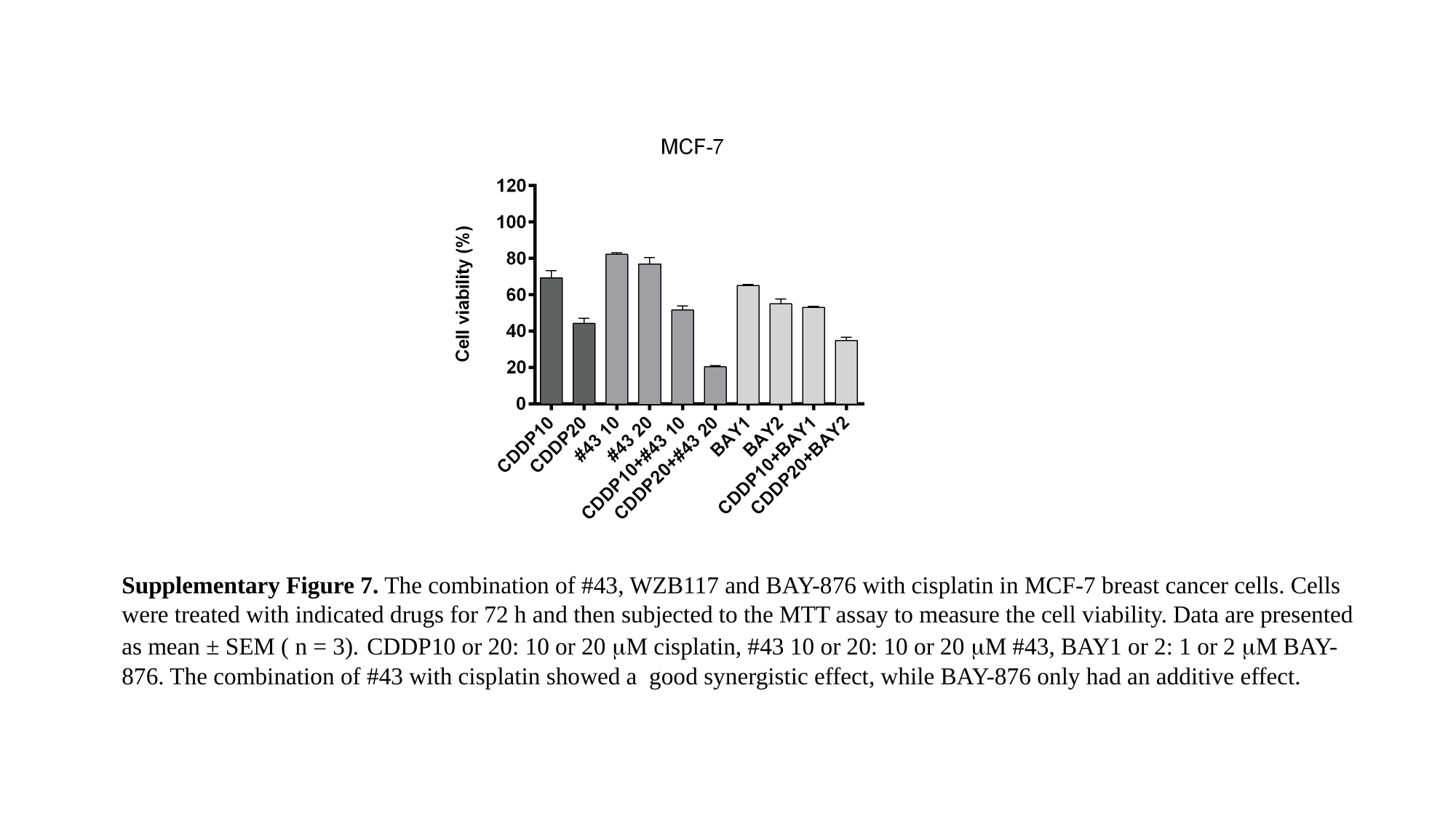

Supplementary Figure 7. The combination of #43, WZB117 and BAY-876 with cisplatin in MCF-7 breast cancer cells. Cells were treated with indicated drugs for 72 h and then subjected to the MTT assay to measure the cell viability. Data are presented as mean ± SEM ( n = 3). CDDP10 or 20: 10 or 20 μM cisplatin, #43 10 or 20: 10 or 20 μM #43, BAY1 or 2: 1 or 2 μM BAY-876. The combination of #43 with cisplatin showed a good synergistic effect, while BAY-876 only had an additive effect.
